# RING1B contributes to Ewing sarcoma development by repressing the Na_V_1.6 sodium channel and the NF-κB pathway, independently of the fusion oncoprotein

**DOI:** 10.18632/oncotarget.10092

**Published:** 2016-06-15

**Authors:** Inmaculada Hernandez-Muñoz, Elisabeth Figuerola, Sara Sanchez-Molina, Eva Rodriguez, Ana Isabel Fernández-Mariño, Carlos Pardo-Pastor, María Isabel Bahamonde, José M. Fernández-Fernández, Daniel J. García-Domínguez, Lourdes Hontecillas-Prieto, Cinzia Lavarino, Angel M. Carcaboso, Carmen de Torres, Oscar M. Tirado, Enrique de Alava, Jaume Mora

**Affiliations:** ^1^ Fundació Institut Hospital del Mar d'Investigacions Mèdiques (FIMIM), 08003-Barcelona, Spain; ^2^ Developmental Tumor Biology Laboratory, Department of Pediatric Hematology and Oncology, Hospital Sant Joan de Déu, 08950-Barcelona, Spain; ^3^ Laboratori de Fisiologia Molecular, Departament de Ciències Experimentals i de la Salut, Universitat Pompeu Fabra, 08003-Barcelona, Spain; ^4^ Department of Pediatric Hematology and Oncology, Instituto de Biomedicina de Sevilla (IBiS), Hospital Universitario Virgen del Rocio/CSIC/Universidad de Sevilla, 41013-Seville, Spain; ^5^ Sarcoma Research Group, Laboratori d'Oncología Molecular, Institut d'Investigació Biomèdica de Bellvitge (IDIBELL), L'Hospitalet de Llobregat, 08908-Barcelona, Spain; ^6^ Present Affiliation: Department of Neuroscience and Biomolecular Chemistry, School of Medicine and Public Health, University of Wisconsin, Madison-53705, USA

**Keywords:** RING1B, Ewing sarcoma, voltage-gated sodium channel, NF-κB, FGFR/SHP2/STAT3

## Abstract

Ewing sarcoma (ES) is an aggressive tumor defined by *EWSR1* gene fusions that behave as an oncogene. Here we demonstrate that RING1B is highly expressed in primary ES tumors, and its expression is independent of the fusion oncogene. RING1B-depleted ES cells display an expression profile enriched in genes functionally involved in hematological development but RING1B depletion does not induce cellular differentiation. In ES cells, RING1B directly binds the SCN8A sodium channel promoter and its depletion results in enhanced Nav1.6 expression and function. The signaling pathway most significantly modulated by RING1B is NF-κB. RING1B depletion results in enhanced p105/p50 expression, which sensitizes ES cells to apoptosis by FGFR/SHP2/STAT3 blockade. Reduced Na_V_1.6 function protects ES cells from apoptotic cell death by maintaining low NF-κB levels. Our findings identify RING1B as a trait of the cell-of-origin and provide a potential targetable vulnerability.

## INTRODUCTION

Ewing sarcoma (ES) is an aggressive and poorly differentiated tumor, typically arising from bone and soft tissues in children and young adults. It is characterized by reciprocal translocations that result in almost all cases in the fusion of the EWS RNA binding protein 1 (*EWSR1*) to an ETS transcription factor, being *EWSR1*–*FLI1* the most common chimera [1, 2]. ES tumors display a high degree of genomic stability with very few recurrent mutations besides the pathognomonic fusion, and are among the most genetically normal cancers [3–5]. This strikingly unaltered somatic landscape highlights the role of *EWSR1-FLI1* as the unique trigger of the oncogenic transformation in an otherwise yet unidentified cell-of-origin harboring key features that will likely contribute to the eventual development of ES.

Meta-analysis of data coming from *EWSR1-FLI1* gain-of-function approaches revealed that the genes up-regulated by the fusion in heterologous cell systems are more numerous and display more similarities among different experimental models than the genes down-regulated. Since the cell-of-origin of ES remains elusive, gain-of-function models have been carried out expressing the oncogene in a variety of poorly or undifferentiated heterologous cell types. Genome-wide analysis using high-throughput sequencing technologies have identified a plethora of EWSR1-FLI1 direct targets and shown that EWSR1-FLI1 primarily up-regulates gene expression through the interaction with GGAA repeats present in satellite DNA within the genome [6]. In contrast, data obtained by depleting EWSR1-FLI1 in ES cells revealed that many more genes resulted down-regulated by the fusion oncogene than up-regulated, suggesting that gene repression may be more prevalent than transcriptional activation [7]. However, many of these EWSR1-FLI1 repressed targets are divergent and highly dependent on the cellular background [8]. Since EWSR1-FLI1 directly binds to promoters of a small subset of repressed targets [7], the lack of consistency among the different sets of repressed genes is likely due to a variety of both direct and indirect mechanisms used by EWSR1-FLI1 for gene silencing.

EZH2 is a direct EWSR1-FLI1 target that belongs to the Polycomb (PcG) family of epigenetic regulators and blocks endothelial and neuro-ectodermal differentiation of ES cells [9]. PcG proteins form two major families of complexes, the Polycomb-repressive complex (PRC) 1 and 2. PRC2 comprises EED, SUZ12 and EZH2, which catalyzes the K27 trimethylation of histone H3 (H3K27me3). Mammalian PRC1 includes BMI1, MEL18, and RING1B, which catalyzes H2A K119 ubiquitination (ubH2K119) [10, 11]. PRC1 and PRC2 mostly differ in their genomic localization with a small subset of PRC1 co-localizing with H3K27me3. Adding complexity, six major groups of PRC1 subcomplexes with specific developmental functions and mutually exclusive PRC1 subunits have been described, being RING1B the unique common feature [12]. Importantly, it has recently been reported that RING1B catalytic activity results in gene repression, consistent with the classic repressive function of the Polycomb complexes, whereas catalytic-independent association of RING1B with UTX, an H3K27 demethylase, and p300, leads to transcriptional activation [13].

Despite the important role of the epigenetic landscape in ES, studies addressing the PcG contribution to ES tumorigenesis have been restricted to EZH2 and BMI1. Here we investigate the expression and function of RING1B in ES, a protein with unique abilities among the PcG family of epigenetic regulators.

## RESULTS

### Ewing sarcoma tumors express high levels of RING1B

**Figure 1:**
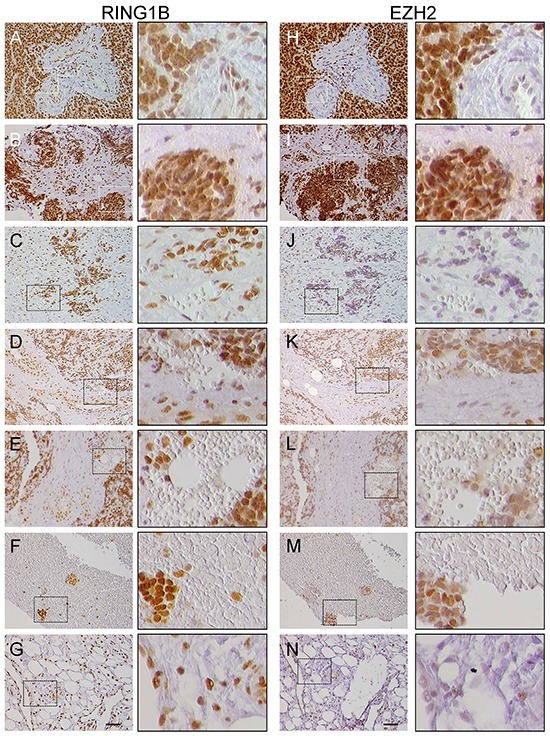
RING1B and EZH2 expression in primary Ewing sarcoma (ES) tumors Immunohistochemistry analysis of RING1B (A-G) and EZH2 (H-N) expression in serial sections of primary ES tumors. Right columns, higher magnifications of the left panel fields (scale bars 50μm). **A, B, H, I.** samples with intense and homogeneous RING1B and EZH2 staining; **C, D, E, J, K, L.** hemorrhagic samples with intense RING1B and low EZH2 staining. Blood lakes can be extensively observed in these tumors; **F, M.** blood clots in an ES sample; **G, N.** adipose tissue in an ES sample.

ES tumors express high EZH2 mRNA levels [9]. To better characterize PcG expression we analyzed EZH2 and RING1B protein expression in ES primary tumors. EZH2 was detected in all the tumor samples, most of them with variable EZH2 expression patterns (Figure 1, right). Particularly poor EZH2 expression was found in largely hemorrhagic tumors, blood clots and tumors infiltrating the adipose tissue (Figure 1, J-N). In contrast, RING1B was highly expressed and uniformly distributed throughout the tumor in most samples, reaching the maximum score (Figure 1, left; Supplementary Figure S1A). Of note, RING1B was expressed in endothelial cells of tumor blood vessels and in the adipose tissue (Figure 1C, 1G), whereas RING1B expression was observed in sparse cells of blood clots (Figure 1F). In these tissues EZH2 expression was low. Importantly, RING1B expression in ES was found to be significantly higher than in other developmental tumors such as rhabdomyosarcoma, synovial sarcoma and Wilms tumor (Supplementary Figure S1B).

Since it has been postulated that neural crest-derived progenitors could be the cell-of-origin [14] and ES tumors display a continuum of different degrees of neural differentiation [15], RING1B expression was also investigated in neural tissues. Moderate RING1B expression was found in cerebellum and peripheral sympathetic ganglia, whereas low or no expression was found in the brain and spinal cord (Supplementary Figure S1C). In addition, RING1B could not be detected in undifferentiated neuroblastic aggregates of the normal fetal adrenal medulla, a derivative of the embryonic neural crest (Supplementary Figure S1C).

Next we analysed the expression of BMI1 and MEL18, two PRC1 proteins. Some specimens displayed low BMI1 and MEL18 levels (Supplementary Figure S2A and S2B), accordingly to reported BMI1 expression in many, but not all, ES primary tumors [16]. In embryonic neural tissues BMI1 expression pattern resembled that of RING1B, with low to moderate expression in most of the embryonic neural tissues (Supplementary Figure S2C).

### RING1B expression is independent of EWSR1-FLI1

**Figure 2:**
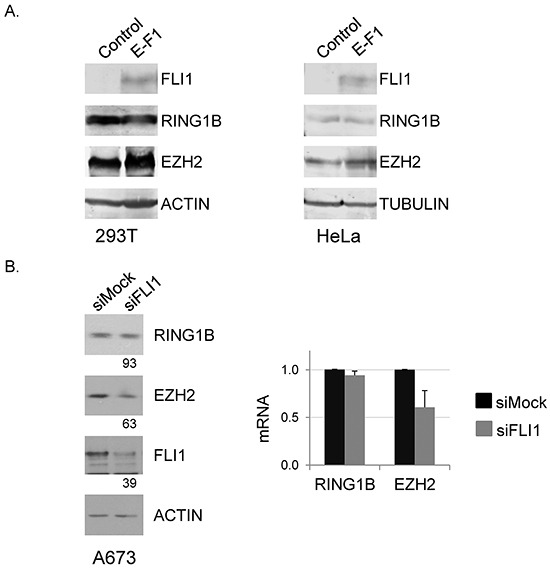
RING1B expression is independent of *EWSR1-FLI1* **A.** *EWSR1-FLI1* was ectopically expressed in 293T (left panel) and HeLa (right panel) cells and RING1B and EZH2 levels were determined by western blot. Actin and Tubulin, loading controls. **B.** A673 cells were transiently transfected with mock or FLI1 short interfering RNA (siRNA) and RING1B and EZH2 levels were determined 48 hours later by western blot (left panel) and qRT-PCR (right panel). Numbers at the bottom represent protein band intensities normalized to Actin and relative to control cells, as described in “Methods”. Graph shows mean ± SD of three replicate samples from one representative experiment out of three.

To test whether RING1B expression was modulated by the oncogene, EWSR1-FLI1 was introduced in 293T and HeLa cells. In both cell lines EZH2 expression was enhanced, as previously reported [9], whereas RING1B levels were not affected (Figure 2A). Conversely, down-regulation of EWSR1-FLI1 in A673 cells resulted in diminished EZH2 levels, but RING1B levels remained unaffected (Figure 2B). Accordingly, endogenous EWSR1-FLI1 binds specifically to a conserved ETS recognition sequence of the EZH2 promoter [17]. In contrast, the genomic region of RING1B was not reported to be an EWSR1-FLI1-direct binding domain [6, 18] nor RING1B was a differentially expressed gene in EWSR1-FLI1-depleted cells [19, 20]. We suspected that EWSR1-FLI1-independency of RING1B expression might be reflecting the high levels of expression of the cell-of-origin where the oncogenic translocation occur, likely regulating key functions of this cell. Therefore, we decided to study the role of RING1B in ES cells by depleting RING1B protein levels.

### Gene expression profile in RING1B-depleted ES cells is unique and enriched in genes functionally involved in hematological development

**Figure 3:**
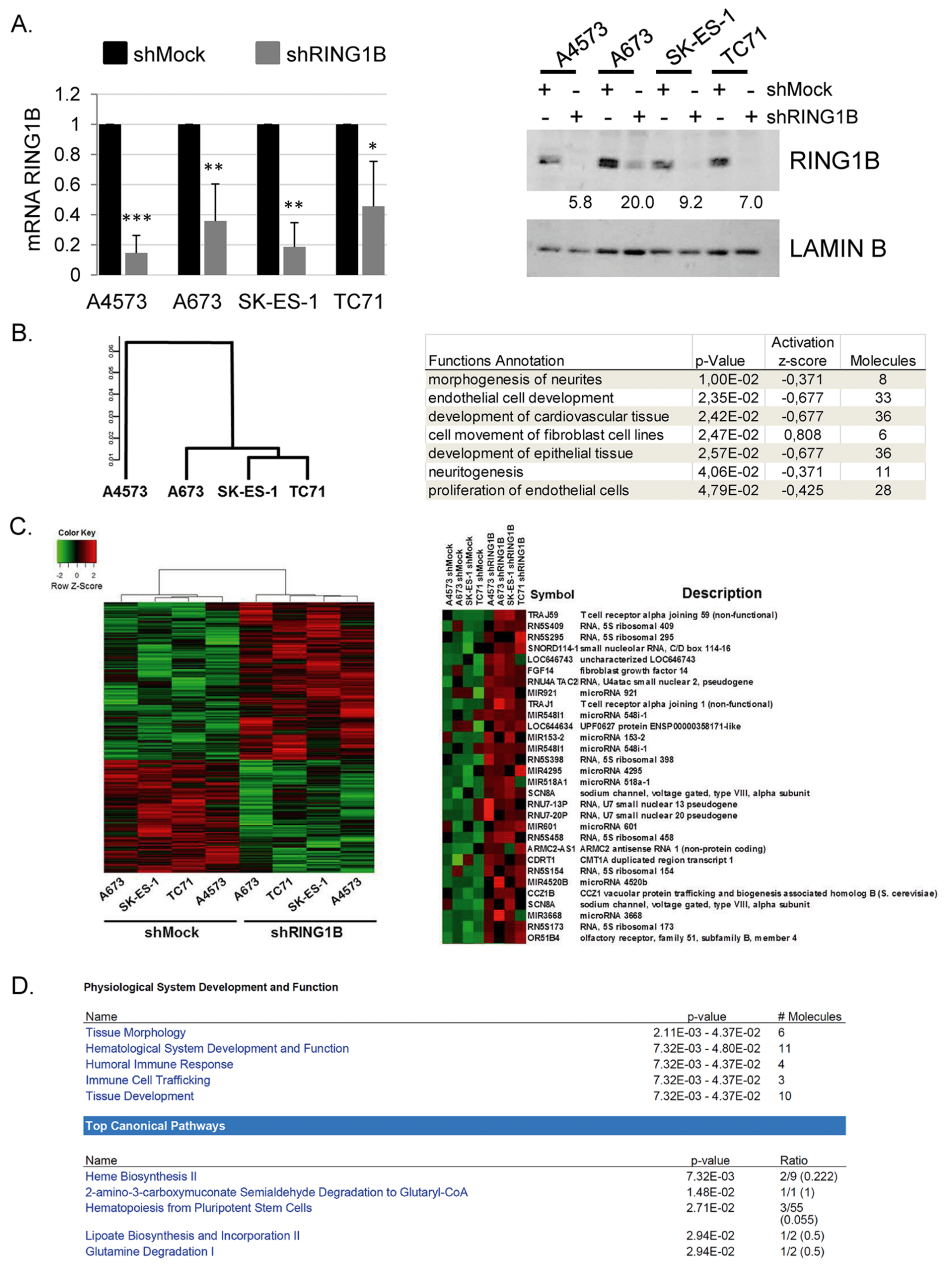
Characterization of the transcriptome in RING1B-depleted ES cells **A.** Efficiency of stable Ring1B knockdown (short hairpin, sh) in ES cells determined by qRT-PCR (left panel) and western blot (right panel). *, P < 0.05; **, P < 0.01; ***, P < 0.005. Numbers at the bottom, RING1B expression normalized to Lamin B and relative to RING1B levels in each control cell line. **B.** Cluster dendrogram derived from array expression data of the four ES parental cell lines (left panel) and functions of the genes differentially regulated in the A4573 cell line versus A673/SK-ES-1/TC-1 cell lines, identified by the Ingenuity Pathway Analysis (IPA) software (right panel). **C.** Primary heat map of genes differentially expressed between shMock and shRING1B cells. Right panel, heat map depicting the first 30 annotated and up-regulated genes in shRING1B cells. **D.** Top functional annotations enriched among RING1B-regulated genes, identified by IPA software. Array data deposited at the Gene Expression Omnibus (GEO, National Center for Biotechnology Information) with accession number GSE71007.

To identify genes whose expression is regulated by RING1B in ES tumors we analyzed four different ES cell lines stably depleted of RING1B by short hairpin RNA (shRNA) (Figure 3A). Gene expression analysis followed by unsupervised hierarchical clustering of the most variably expressed genes stratified parental cell lines into one main group formed by A673, SK-ES-1 and TC71 and segregated the A4573 cell line (Figure 3B). Of note, A4573 cells also display low BMI1 protein levels [21].

Despite substantial differences in A4573 gene expression profile, linear models for microarrays (LIMMA) analysis was performed using the four cell line data in an attempt to identify common traits of RING1B gene regulation in ES. Logarithmic fold change for each shRING1B/shMock sample pair revealed that RING1B depletion resulted in altered expression of 1430 sequences (Figure 3C). Among them, 523 corresponded to annotated genes, 259 up-regulated (49%) and 264 down-regulated (51%), a ratio that parallels that of RING1B-transcriptionally regulated genes in melanoma [13]. The genes differentially expressed in RING1B-depleted cells were then analyzed at the functional level using the IPA software. This analysis revealed “tissue morphology” and “hematological system development and function” as the top two biological functions altered, while the top canonical pathways identified included “heme biosynthesis” and “haematopoiesis from pluripotent stem cells” (Figure 3D).

To compare RING1B with other PcG proteins, BMI1 was depleted in A673 and TC71 cells (Supplementary Figure S3A). Gene expression analysis revealed that BMI1 or RING1B depletion resulted in 734 and 838 differentially expressed sequences, respectively (Supplementary Figure S3B), 196 overlapping, and among these, 70 were annotated sequences (Supplementary Figure S3C). Most genes, however, were differentially regulated by each PRC1 protein in ES cells.

**Figure 4:**
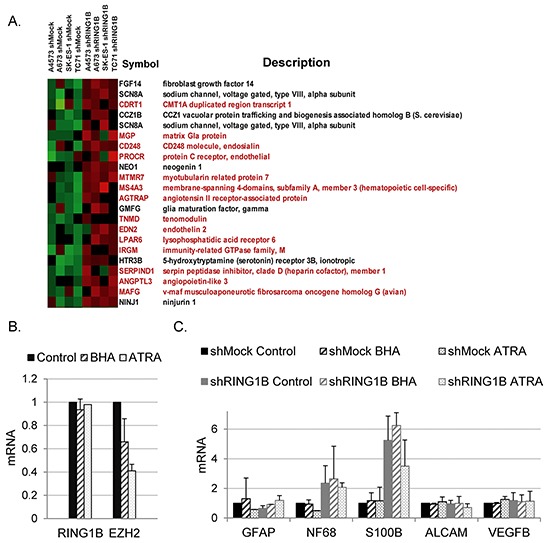
RING1B depletion does not result in global alterations of developmental transcription programs in ES cells **A.** Heat map showing endothelial (red) and neural (black) genes up-regulated in RING1B-depleted cells, identified by manual curation of the microarray annotated sequences. **B.** RING1B and EZH2 mRNA levels determined by qRT-PCR in SK-ES-1 cells treated with differentiating agent butylated hydroxyanisole (BHA) and all trans-retinoic acid (ATRA). **C.** SK-ES-1 cells, either shMock or shRING1B, were treated with BHA or ATRA and mRNA levels of different neural or endothelial genes were analyzed by qRT-PCR. **B and C.** graphs show mean ± SD from two independent experiments performed with three replicate samples.

EZH2 downregulation in ES cells results in the induction of genes important for neural and endothelial differentiation [9]. In contrast, few endothelial and neural genes were identified among the annotated transcripts in RING1B-depleted cells (Figure 4A). Furthermore, RING1B depletion only resulted in enhanced expression of three genes, MS4A3, CDRT1 and TLE4, out of the most significantly EZH2 downregulated genes in ES cells [9] (Figures S4, S5).

All-trans retinoic acid (ATRA)-induced neural stem cell differentiation results in EZH2 down-regulation and decreased binding of EZH2 to ATRA-inducible target genes [22]. Particularly important for ES biology is the differentiation blockade that EZH2 imposes in ES cells, which can undergo neuroectodermal differentiation with butylated hydroxyanisole (BHA) only upon EZH2 depletion [9]. ATRA and BHA treatment of SK-ES-1 cells resulted in reduced EZH2, but not RING1B, mRNA levels (Figure 4B). Furthermore, neither ATRA nor BHA were able to induce differentiation in shRING1B cells since mRNA levels of neural and endothelial differentiation genes remained similar to those of the untreated cells (Figure 4C).

### RING1B regulates the Na_V_1.6 sodium channel in Ewing sarcoma cells

**Figure 5:**
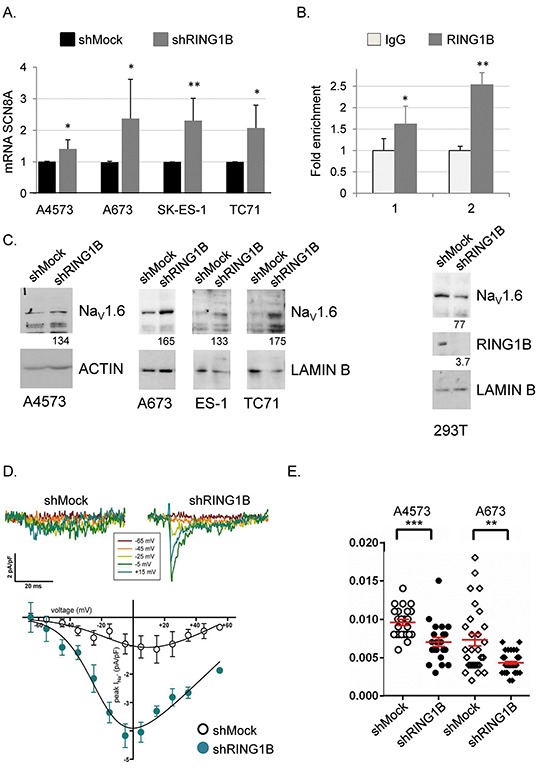
RING1B represses the NaV1.6 channel in ES cells **A.** SCN8A mRNA levels in RING1B-depleted cells determined by qRT-PCR and referred to shMock control mRNA levels. *, P < 0.05; **, P < 0.01. **B.** Relative enrichment of RING1B occupancy at SCN8A promoter, measured by ChIP-qPCR. Graph displays percentage of RING1B recruitment to two different SCN8A promoter amplicons, referred to enrichment from the IgG control, set as 1. Data express mean ± SD of three independent experiments. *, P < 0.05; **, P < 0.01. **C.** Na_V_1.6 protein levels in cytoplasmic (A4573) and total (A673, SK-ES-1, TC-71, 293T) extracts from control or RING1B-depleted cells, analyzed by immunoblot. Numbers at the bottom, band intensities normalized to Actin or Lamin B and relative to control cells. **D.** Representative current traces obtained from shMock and shRING1B SK-ES-1 cells clamped at −90 mV and pulsed for 40 ms to the indicated depolarizing voltage (top) and average current density-voltage (I-V) relationships for shMock (open circles, n = 5) and shRING1B (filled cyan circles, n = 5) cells (bottom). Fast-activating inward currents with both transient and persistent components, consistent with Na_V_1.6 currents, were only observed in shRING1B SK-ES-1 cells (peak current was 4.2 ± 0.4 pA/pF) and the corresponding V1/2 act, kact and Vrev values after fitting the I-V curve to a Boltzmann equation (see Methods for further details) were (in mV): −21 ± 4.1 mV (as reported for Na_V_1.6 channels heterologous expression in the ND7/23 neuronal cells, either in the absence or presence of FGF14 (54;61)), 10.8 ± 2.1 and 84.5 ± 16.3, respectively. **E.** Cell migration speed measurements (in micron/sec) during wound healing from shMock (open) and shRING1B (black filled) A4573 and A673 cell lines (obtained in three different experiments). Symbols represent individual cells. Red bars depict mean ± SEM speed. Suppressing RING1B expression, which correlates with increased SCN8A expression, decreased cell speed in A4573 and A673 cells. **, P<0.01; ***, P<0.001.

FGF14 and SCN8A were among the top 30-upregulated annotated sequences upon RING1B depletion (Figure 3C), and these increases where validated by qRT-PCR in RING1B-depleted cells (Figures 5A, S6A). *SCN8A* encodes the α subunit of the voltage-gated sodium channel type VIII, or Na_V_1.6. This protein forms a sodium-selective channel essential for rapid membrane depolarization in response to voltage differences in excitable neurons. FGF14 is a member of the intracellular fibroblast growth factor subfamily (iFGF) that co-localizes and interacts with the Na_V_ channel α subunits, being Na_V_1.6 the most sensitive to FGF14 modulation [23]. While RING1B was not detected at the FGF14 promoter (data not shown), it was found to bind to the promoter region of the SCN8A gene in ES cells (Figure 5B). RING1B depletion using an independent shRNA construct [11] confirmed that *SCN8A* is repressed by RING1B in ES cells (Supplementary Figure S6B). SCN8A released expression in RING1B-depleted cells resulted in enhanced Na_V_1.6 protein levels (Figure 5C). In contrast, RING1B depletion in 293T did not affect Na_V_1.6 levels (Figure 5C) while in neuroblastoma SK-N-SH cells caused subtle increases in SCN8A mRNA levels (Figures S6C), suggesting that RING1B regulation of the Na_V_1.6 channel is specific of ES cells.

Next we investigated Na_V_1.6 channel regulation by other PcG proteins. BMI1 depletion resulted in SCN8A down-regulation while EZH2 depletion lead to minor changes in SCN8A mRNA levels (Supplementary Figure S7). Furthermore, treatment with the histone methylation inhibitor DZNep did not affect SCN8A mRNA levels (Supplementary Figure S7D). All these data suggest that Na_V_1.6 channel repression in ES is specifically set by RING1B.

To check the functional impact of the enhanced expression of Na_V_1.6 upon RIING1B depletion we used the voltage-clamp technique in the whole-cell configuration to record voltage-gated sodium currents. In response to depolarizing pulses, significant fast-activating and fast-inactivating inward sodium currents, with a persistent component (consistent with Na_V_1.6 electrophysiological properties [24, 25]), were only recorded in shRING1B cells but not in shMock control cells (Figure 5D). Since Na_V_1.6 knockout in dorsal-root ganglion cells results in migration towards the ventral region and acquisition of a parasympathetic phenotype [26] we investigated the migratory abilities of ES cells. A4573 and A673 control cells displayed migratory abilities that were attenuated in RING1B depleted cells (Figure 5E). Since RING1B depletion affects the expression of very few neural genes and did not alleviate ES cells from their neural differentiation blockade, this result suggests that targeted Na_V_1.6 repression might be of particular relevance for ES biology.

### RING1B modulates the NF-κB pathway in Ewing sarcoma cells

**Figure 6:**
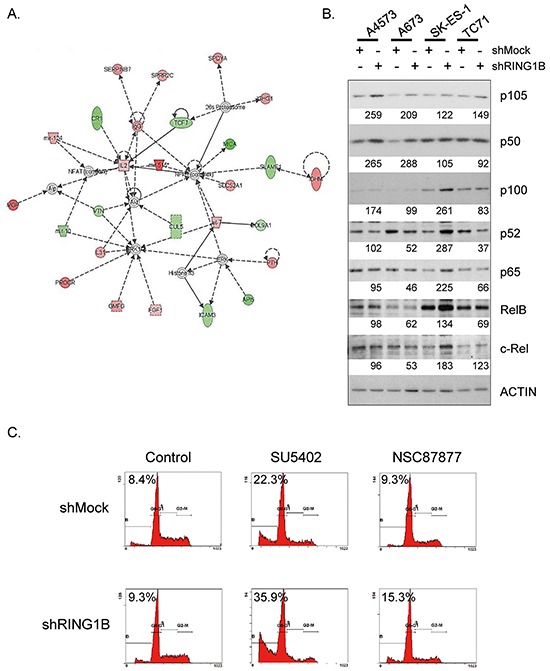
RING1B represses NF-κB in ES cells **A.** Top-scoring multigene network associated by the IPA software to microarray expression data in RING1B-depleted cells. Overexpressed genes, red; downregulated genes, green. **B.** Levels of NF-κB proteins, detected by immunoblot performed using total extracts of shMock and shRING1B cells. Numbers at the bottom, band intensities normalized to Actin and relative to their own control cells. **C.** Flow cytometry analysis of A673 cells treated with 25 μM SU5402 or 50 μM NSC87877 for 24h. Percentage of cells in the sub-G1 region is indicated. DNA histograms of 10,000 propidium iodide stained cells.

To identify molecular pathways affected by RING1B-modulated targets we conducted IPA. On the basis of the four RING1B-depleted cell line signature, the IPA analysis revealed NF-κB as the most significantly altered node (Figure 6A). To explore this pathway we performed western blot analyses of the five members of the NF-κB transcription factor family: NF-κB1 (p50 and its precursor p105), NF-κB2 (p52 and its precursor p100), c-Rel, RelA/p65 and RelB. Levels of p105 and p50 were enhanced to variable degrees in shRING1B cells when compared to their corresponding shMock controls (Figure 6B). RING1B depletion using an independent shRING1B also resulted in consistently enhanced p50 levels (Supplementary Figure S8A). RING1B-depletion in non-ES cells like 293T and SK-N-AS (neuroblastoma) displayed inconsistent changes in NF-κB1 levels (Supplementary Figure S8C).

**Figure 7:**
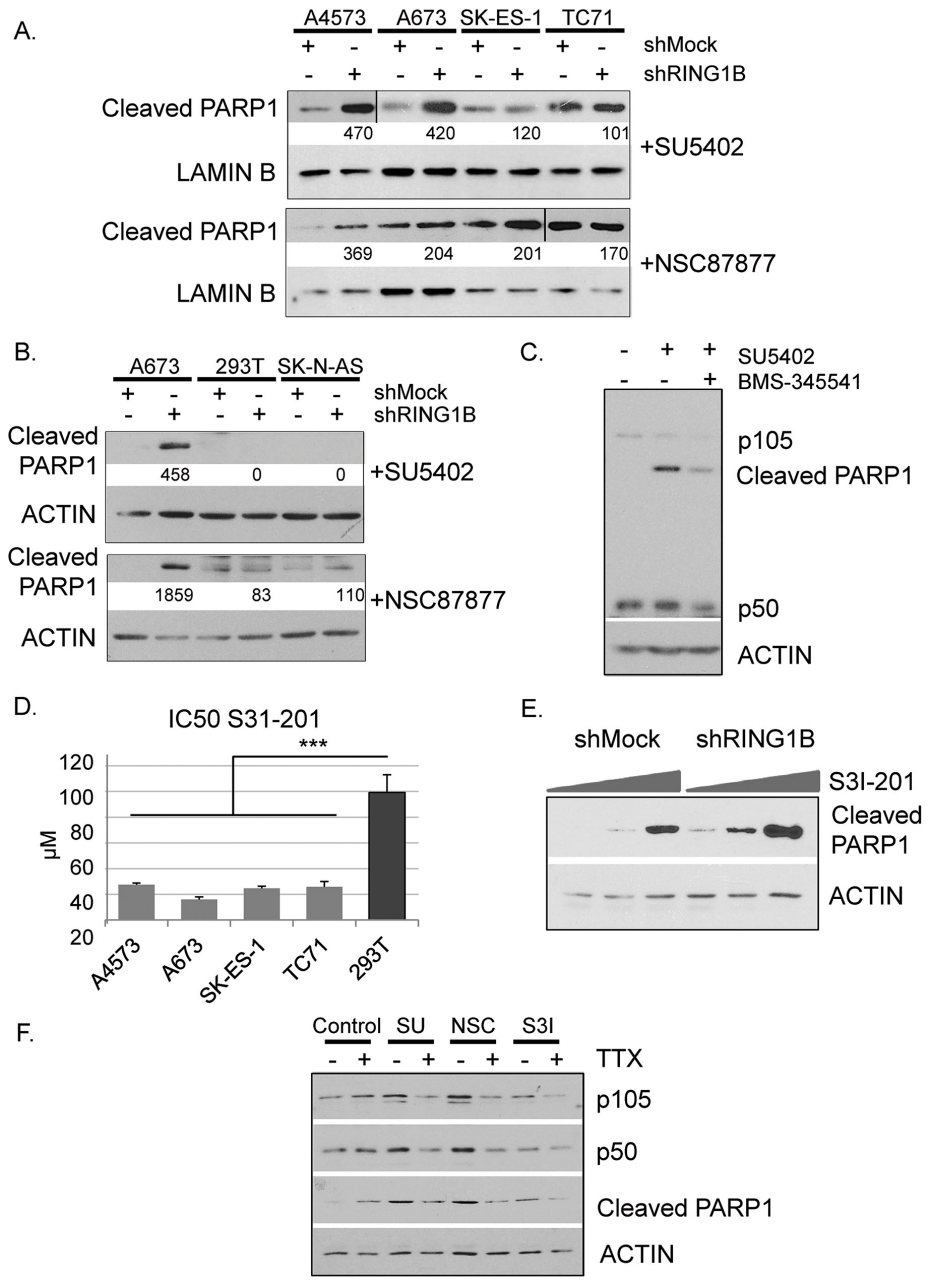
RING1B down-regulation sensitizes ES cells to undergo NF-κB-mediated apoptosis induced by FGFR signaling blockade **A.** Immunoblot detection of cleaved PARP1 in shMock and shRING1B cells treated with 25μM SU5402 or 50 μM NSC87877 for 24h. **B.** Immunoblot detection of cleaved PARP1 in 293T embryonic and SK-N-AS neuroblastoma cells, treated as above. Numbers at the bottom, band intensities normalized to Actin or Lamin B and relative to their own control cells. **C.** Immunoblot detection of NF-κB1 (p105 and processed form p50) and cleaved PARP1 in A673 shRING1B cells after 24h with or without 25μM SU5402 and the inhibitor of the IκB kinase (IKK)α/β BMS-345541 at 300nM. **D.** Half maximal inhibitory concentration (IC50) values for S31-201 in ES and 293T cells, measured after 72h treatment and expressed as mean ± SD (n=3). ANOVA-Bonferroni post-hoc test, *** <0.001. **E.** Cleaved PARP1 in shMock and shRING1B A673 cells, after 24h treatment with the STAT3 inhibitor S3I-201 at 25μM, 50μM and 100μM. **F.** Levels of the NF-κB subunits p105 and p50, and cleaved PARP1 in shRING1B A673 cells, after 24h treatment with 25μM SU5402, 50 μM NSC87877 or S3I-201 at 50μM in the absence or the presence of 1μM Tetrodotoxin (TTX).

Since the NF-κB pathway is a regulator of stress response, next we checked the sensitivity of ES cells to challenging conditions. A673 cells were subjected to a panel of chemical inhibitors and cell viability was tested by flow cytometry using propidium iodide staining. Two drugs induced significant apoptotic cell death: SU5402 and NSC87877. Whereas SU5402 irreversibly blocks FGF and VEGF tyrosine kinase receptors [27], NSC87877 specifically inhibits SHP2 [28], a tyrosine phosphatase that participates in the regulation of FGFR signalling [29]. Interestingly, RING1B-depleted cells treated with these inhibitors showed enhanced sub-G1 peaks when compared to shMock cells (Figure 6C). To better characterize this effect, the four ES cell lines, either shMock or shRING1B, were treated with the two inhibitors. NF-κB1 levels in shRING1B cells remained elevated compared to their own shMock controls regardless of the inhibitor treatment (Supplementary Figure S8B). SU5402 and NSC87877 treatments resulted in enhanced cleaved PARP1 levels in shRING1B cells when compared to controls (Figure 7A), indicating that RING1B depletion sensitizes ES cells to the apoptosis induced by the FGFR-SHP2 blockade. In contrast, RING1B depletion in 293T and SK-N-AS cells did not affect cleaved PARP levels (Figure 7B). IκB kinase (IKK) α/β inhibition with BMS-345541 attenuated SU5402-induced PARP1 cleavage in A673 shRING1B cells, further suggesting that NF-κB is pivotal for RING1B-mediated sensitivity to apoptosis in ES cells (Figure 7C).

Activation of FGFRs results in the stimulation of several signaling pathways including STAT3. Therefore, we investigated the sensitivity of ES cells to the STAT3 inhibitor S31-201. As shown in Figure 7D, ES cells viability was affected at significantly lower concentrations of S31-201 than 293T cells. RING1B depletion further sensitized ES cells to S3I-201, as shown by the appearance of cleaved PARP1 at lower inhibitory concentrations compared to control cells (Figure 7E).

Increases in intracellular sodium through voltage sensitive sodium channels can contribute to apoptotic cell death [30]. Therefore we asked whether the enhanced Na_V_1.6 functionality in RING1B depleted cells increased the sensitivity of ES cells to apoptosis by selective blocking of the voltage-sensitive sodium channel with Tetrodotoxin (TTX). A673 shRING1B cells treated with SU5402, NSC87877 or S31-201 and cultured in the presence of TTX showed decreases in p105 and p50 levels and lower cleaved PARP levels than cells cultured in the absence of TTX, suggesting that RING1B repression of Na_V_1.6 also contributes to protect ES cells from apoptotic cell death by maintaining low NF-κB levels (Figure 7F).

## DISCUSSION

Recent next-generation sequencing studies have revealed the simplicity of the ES genome [1;3-5] highlighting the critical importance of the *EWSR1–ETS* fusion event. The mechanism by which *EWSR1-FLI1* contributes to tumorigenesis is complex since the fusion oncogene affects the cell in many different ways. The best known function of the EWSR1-FLI1 protein is that of an aberrant transcription factor. However, other EWSR1-FLI1 properties including RNA binding, RNA splicing and protein-protein interactions are increasingly being recognized as fundamental to understand the plethora of effects induced by the fusion oncogene. Equally important has been the recognition of the critical role of the cellular context for EWSR1-FLI1 activity. In normal murine and human fibroblasts, *EWSR1-FLI1* by itself does not transform cells; instead, it results in cell cycle arrest [31]. Related to this is the fact that it has been very difficult to establish an appropriate animal model by introduction of *EWSR1-FLI1* chimeras, suggesting that *EWSR1-FLI1* is not sufficient to define the origin of ES. Finding universal features for a cell-of-origin to tolerate *EWSR1-ETS* chimeras would be extremely helpful to understand ES tumorigenesis. Here, we show that RING1B is highly and universally expressed in primary ES tumors. We also show that RING1B expression is not a direct target of the fusion oncogene and that RING1B levels are not affected by chemically induced differentiation of ES cells. Furthermore, we demonstrate that RING1B regulates, independently of the fusion oncogene, critical pathways that may reflect the biology underlying the cell of origin.

Chromatin repressive complexes like the PcG protein complexes PRC1 and PRC2 are essential mediators of stemness and critical contributors to cancer pathogenesis by suppressing the expression of tumor-suppressor genes and developmental regulators in a context-dependent, cell-type-specific manner [32]. Our data show an intense and universal expression of RING1B in ES primary tumors, in contrast with other PRC proteins which are selectively expressed in some but not all ES tumoral cells. Indeed BMI1 expression is variable among both primary tumors and cell lines in our experience and in previously published studies [21] and EZH2 expression is heterogeneous in most of ES primary tumors. A particularly low EZH2 expression was found in hemorrhagic areas and blood lakes and in tumors infiltrating the adipose tissue. Blood lakes are a striking feature of ES first recognized by James Ewing, which led him to describe the tumor as an endothelioma [33]. Van der Schaft and colleagues described an abundant presence of blood lakes and vascular-like tube formations, suggesting a contribution of the blood lakes to circulation recalling vasculogenic mimicry [34], as had previously been described in melanoma [35]. The lower expression of EZH2 in the blood lakes in our study is consistent with the inhibitory function of EZH2 on endothelial differentiation [9]. In these cells RING1B retains a relatively high level of expression suggesting that RING1B could be important in an endothelial cell context. In this regard, it is notable that NF-κB has been shown to be crucial for endothelial cell fate determination and that NF-κB activation results in apoptosis in endothelial cells [36]. Similarly, we here report that, in shRING1B ES cells, enhanced NF-κB levels are detrimental and sensitize ES cells to apoptosis. Our results also help to explain previous studies reporting very low NF-κB activity in ES [37–39] and suggest that NF-κB is to be repressed in the ES cell-of-origin because elevated NF-κB levels in such cells result in apoptosis. According to our data, the ES cell-of-origin displays traits of endothelial-precursor cell biology.

FGF receptors (FGFRs) include a family of high- and low-affinity bFGF receptors. bFGF, a ubiquitously expressed growth factor that affects a broad spectrum of developmentally regulated cellular responses, induces cell death in ES [40]. Since bFGF has a critical role in the commitment of primitive neural cells toward a neuronal phenotype, the induction of apoptosis in ES was thought to be consistent with the hypothesis that Ewing tumors arise in a primitive neural stem cell. However, the magnitude of bFGF-induced cell death was heterogeneous suggesting that ES can arise from primitive cells at various stages and that the expression of different growth factors and hormones by Ewing tumors may modulate the effect of bFGF [41]. Our results suggest that RING1B regulates the apoptotic response of FGFR activation in ES cells and thus it could be the modulator element proposed in previous publications between extracellular signaling (hormones and growth factors) and cell type response. More recently, the FGF/FGFR signaling has been found to play a vital role in the development and maintenance of bone growth at growth plates [42]. Since we provide evidence of an active FGF/SHP2/STAT3 pathway in the potential Ewing cell-of-origin, a putative relationship with normal bone growth and development deserves further investigation.

**Figure 8:**
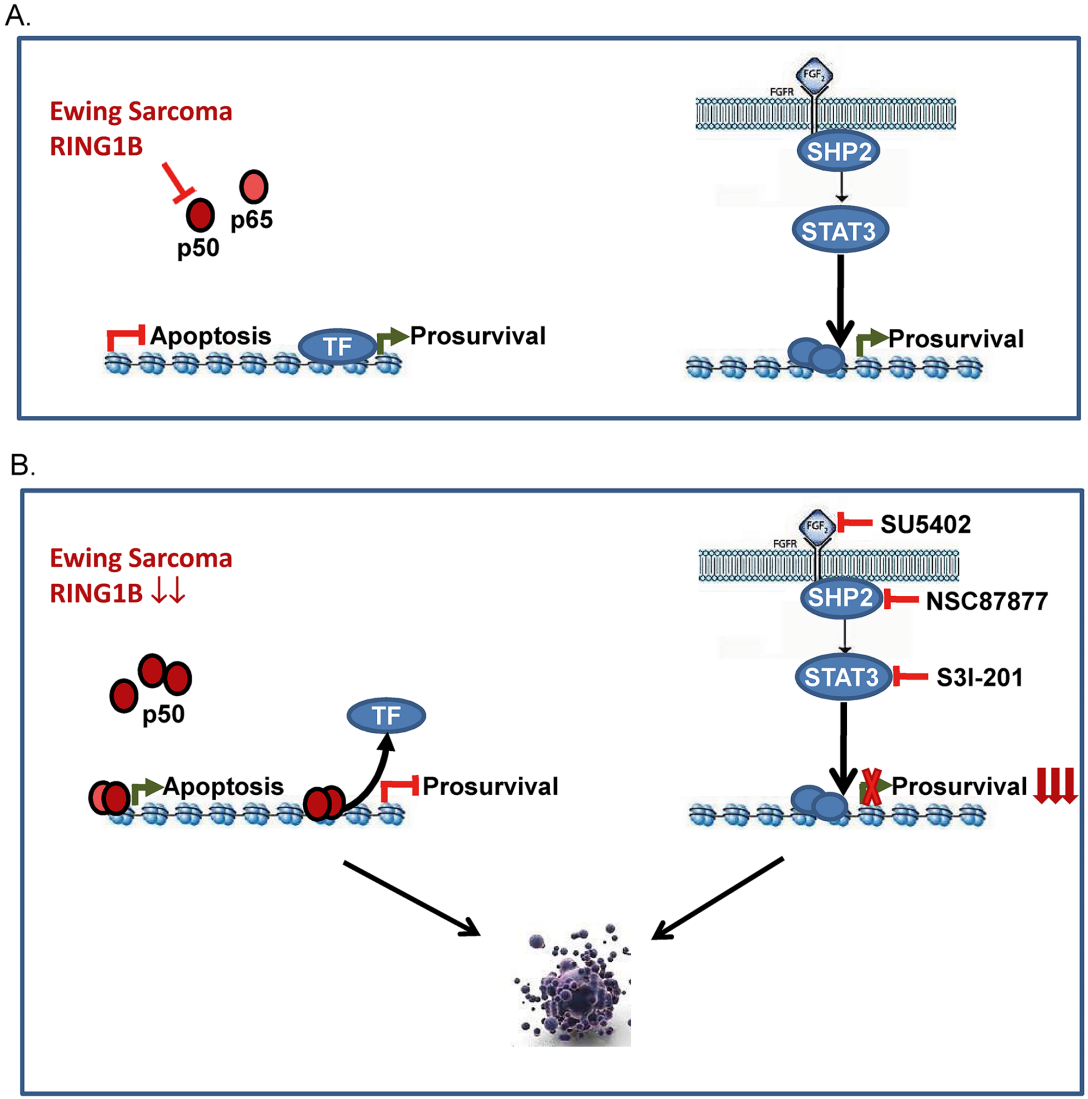
Proposed model for RING1B-dependent antiapoptotic function in ES **A.** According to our results we propose an endothelial linage of the ES cell-of-origin where bFGF/VEGF are specially active and, upon binding to their receptors, activate a signaling cascade that transmits a nuclear signal prompting target genes to activate cell growth. As reporter for an endothelial progenitor cell (EPC) characteristic biology, proapoptotic genes remain inactive in cells with low NF-κB levels tightly controlled by RING1B in ES cells. **B.** Similar to EPC when treated with antiangiogenic factors, RING1B depletion in ES cells results in NF-κB activation, which binds to proapoptotic gene promoters and/or displaces transcription factors from prosurvival gene promoters. In this situation ES cells are highly sensitive to FGF/SHP2/STAT3 blockade (adapted from [36]).

STAT3 is known to drive pathways regulating normal cell growth, which result in tumorigenesis when deregulated [43]. Reportedly, 50% of ES primary tumors express activated/phosphorylated STAT3 [44, 45] and germline mutations of Protein tyrosine phosphatase delta (PTPRD, a STAT3 phosphatase) have recently been described in three ES patients [46]. Aberrant activation of STAT3 signaling participates in the initiation, development and progression of human cancers via induction of STAT3 downstream genes that encode antiapoptotic proteins, cell cycle regulators and angiogenic factors such as Bcl-2, Cyclin D1 and VEGF [47]. Also, increased macrophage infiltration and tumor microvascular density have been noted in tumors from ES patients with poor prognoses and tumor associated macrophages are known to express high concentrations of cytokines that lead to aberrant activation of the JAK/STAT3 pathway. In addition, EWSR1-FLI1 inhibition in ES cells resulted in the secretion of soluble factors, such as IL6, that activate STAT3 in bystander (non-targeted) ES cells, protecting them against apoptosis [48]. Since NF-κB modulates the expression of a variety of cytokines, release of NF-κB inhibition upon RING1B knockdown could result in a feed forward loop of STAT3 activation and cytokine production. This adaptive response suggested that combination therapy with STAT3 inhibitors may increase the efficacy of targeted therapeutics in ES. Reports on JAK/STAT3 inhibitors have shown *in vitro* and *in vivo* activity against ES. For instance, Thiele *et al.* have shown the antitumoral activity of the JAK1/2 inhibitor AZD1480 blocking endogenous constitutive and cytokine-induced activation of STAT3 *in vitro* and suppressing the growth of ES xenografts *in vivo* [49]. Our results showing higher sensitivity to STAT3 inhibition in RING1B-depleted cells suggest that combined inhibition of RING1B and STAT3 could generate enhanced antitumor effects against ES (proposed model in Figure 8). Inhibition of STAT3 activity is well-known to enhance chemosensitivity of multiple tumor types to a number of different cytotoxic agents or other targeted agents; therefore multiple combinations of target inhibition of key pathways for ES like RING1B and STAT3 together with conventional chemotherapy could represent a novel approach for ES patients.

The role of PcG increasing the chances for the cancer cell to survive in hostile microenvironments has recently been shown in ES and other tumors by Lawlor *et al.* [50]. They described KCNA5 repression by BMI1, and the impairment in the execution of hypoxia-induced apoptotic cell death. In this study we show that Na_V_1.6 regulation in ES is specific to RING1B, not being shared with other PcG proteins. RING1B represses the expression and function of this sodium channel, normally expressed in central neurons, dorsal root ganglia, peripheral neurons, heart, and glial cells. The selectivity of this regulation by RING1B is striking because many other neural genes are actively induced by the *EWSR1-ETS* fusion, globally resulting in a neural-like phenotype of the ES cell. Together with the well-known migration blockade caused by EWSR1-FLI1 in the chicken model [51] and our results would suggest co-regulation of the cellular migratory properties of ES cells by RING1B and the fusion oncogene.

The origin of ES has been an enigma since the first case was reported in 1921 [33]. Primitive neural crest cells, hematopoietic cells, and muscle cells as well as mesenchymal stem cells (MSC) have been considered possible cells of origin [52, 53]. Histologically, ES has a certain resemblance to primitive neuroectodermal cells [54]. Early neural markers are present in some tumors and ultrastructural features of neural cells can also be observed in some cases [15, 55, 56]. Furthermore, ES cells can be induced to differentiate in the laboratory towards the neural lineage [57]. Conversely, introduction of *EWSR1-FLI1* into neuroblastoma cells has been shown to make the cells acquire characteristics of ES [58]. EWSR1-FLI1 has been shown to induce neuroectodermal differentiation and up-regulate a number of genes associated with early neural differentiation [59]. Altogether, these findings raise the possibility that the neuroectodermal characteristics of the ES cell might be a direct result of *EWSR1-FLI1* expression and not necessarily reflect a feature of the cell-of-origin.

An alternative hypothesis is that ES derives from a MSC. Although cranial bones develop from mesenchymal condensation of neuroectoderm [52], the long bones of the limbs originate from mesoderm [60] and there may not normally be primitive neuroectodermal cells in that bone. However, bone marrow mesenchymal cells can exhibit some characteristics of neuroectodermal cells [61, 62]. These cells can also be induced *in vitro* to differentiate towards the neural lineage [63, 64]. The term MSC usually implies a cell that has the capacity to differentiate towards various mesodermal lineages. Indeed, EWSR1-FLI1 knockdown revealed that ES cells have some capacity for *in vitro* differentiation towards chondroblastic, osteoblastic and adipocytic lineages [53]. Furthermore EWSR1-FLI1 expression in mesenchymal cells resulted in development of ES tumors [65, 66] and *EWSR1-FLI1* introduction into a population of cells enriched for osteochondrogenic progenitors derived from the embryonic superficial zone of murine long bones revealed a subpopulation of precursor cells that further enhanced EWSR1-ETS–dependent tumor induction [67]. It is yet unclear whether there is a special subfraction of precursor cells that includes the cell of origin of ES, although Stamenkovic and colleagues have reported that primary ES tumors harbor a subpopulation of cells that express CD133 constituting 3-15% of tumor cells that display the plasticity, clonogenicity and tumor-initiating capacity of tumor stem cells [68].

Murine studies have shown that the target cells of *EWSR1-ETS* might be cells of a narrow lineage of MSC and/or of a limited differentiation stage in support of a mesenchymal origin of ES. However, the simple knockdown of EWSR1-FLI1 in tumor cells does not cause them to revert to a normal mesenchymal cell. Constitutive expression of EWSR1-FLI1 protein in embryonic stem cells causes cell death [69], and mice with expression of *EWSR1-FLI1* in the whole body have an embryonic lethal phenotype [70]. Target expression to bone marrow progenitor cells surprisingly produced leukemias [70]. *EWSR1-FLI1* conditional expression in the mesoderm-derived tissues of the limbs resulted in limb shortening, muscle atrophy, osseous dysplasia and other developmental abnormalities [71]. However, sarcomas did not spontaneously form unless the p53 gene was simultaneously mutated [71].

After all, it would seem that the “cell-of-origin” concept for ES may turn out as too simplistic. *EWSR1-FLI1* may be fully transforming only cells that possess the right conditions, which are likely very limited during development. Such a narrow window of target cell emergence would explain the difficulty of inducing tumors in *in vivo* models. We suggest that RING1B is one defining epigenetic precondition for the oncogenic fusion to occur and, at least for some cases, reflects the cellular background of a hemato-endothelial precursor cell as the cell of origin.

## MATERIALS AND METHODS

### Study approval

A written informed consent was received from all patients prior to inclusion of their samples in the Hospital Sant Joan de Déu (HSJD) tumor biobank. Sixteen primary ES specimens were used in the study. Procedures were approved by the Ethical Committee for Clinical Research at HSJD.

### Immunohistochemistry

Immunohistochemical analyses were performed following standard techniques [72]. RING1B and BMI1 antibodies were purchased from Millipore and EZH2 and MEL18 antibodies from Cell Signaling. Immunohistochemical staining was scored for staining intensity and proportion of stained cells by two independent investigators. A semiquantitative histoscore (0×% negative cells + 1×% weakly stained cells + 2×% moderately stained cells + 3×% strongly stained cells) was generated for statistical analysis. This histoscore thus has a range of possible scores between 0 and 300. Statistical analysis was performed with the Wilcoxon signed rank test.

### Gene expression analysis

Microarray analysis, amplification, labeling and hybridizations were performed according to protocols from Ambion WT Expression Kit (Ambion), labeled using the WT Terminal Labeling Kit (Affymetrix), and then hybridized to GeneChip Human Gene 2.0 ST Array (Affymetrix).

### Electrophysiology

Whole-cell voltage-clamp recordings from shMock and shRING1B ES1 cells were obtained using a D-6100 Darmstadt amplifier (List Medical) filtered at 1 kHzand corrected for leak and capacitive currents using the leak subtraction procedure (P/8). Data are expressed as mean ± SEM. For the generation of current-voltage (I-V) curves, peak inward Na^+^ currents were measured from cells clamped at −90 mV and pulsed for 40 ms from −65 mV to +55 mV in 10 mV steps. Single I-V curves at each experimental condition were averaged to obtain the shown I-V curves.

### Flow cytometry

Sub-G1population staining was performed as previously reported [73] and the analysis was performed by FACScan (Coulter).

### Cell viability assay

Cells were seeded at 3000 per well in 96-well culture plates. S3I-201 (STAT3 inhibitor) was added to complete growth medium at concentrations ranging from 1 to 200 μM, respectively. After 72 h, cells were subjected to the ATPlite assay (PerkinElmer, Waltham, MA, USA) and inhibitory concentrations were calculated.

### Statistics

Each experiment was performed in triplicate at least three times unless otherwise indicated. Statistical analysis was performed with Student's t-test. P < 0.05 was considered significant.

Extended methods on cell culture, protein and mRNA analysis, gene expression arrays and electrophysiology are available as *Supplemental Information.*

## SUPPLEMENTAL EXPERIMENTAL PROCEDURES FIGURE AND TABLES


