# Supplementary material for: RING1B contributes to Ewing sarcoma development by repressing the NaV1.6 sodium channel and the NF-κB pathway, independently of the fusion oncoprotein
Source: Oncotarget. 2016 Jun 15;7(29):46283–300. doi: 10.18632/oncotarget.10092 (PMC5216798; doi:10.18632/oncotarget.10092)
Supplement: Supplementary file 1 [file oncotarget-07-46283-s001.pdf]

RING1B contributes to Ewing sarcoma development by repressing the $Na_V1.6$ sodium channel and the NF-κB pathway, independently of the fusion oncoprotein

SUPPLEMENTAL EXPERIMENTAL PROCEDURES

Cell culture

Cells were grown in DMEM or RPMI 1640 (Gibco) in standard conditions. To perform treatments, cells were treated with DNZep (Cayman Chemical), SU5402 (Calbiochem), NSC87877 (Millipore), S3I-201 (Sigma), BMS-345541 (Sigma) and Tetrodotoxin (TTX, Alomone Lab) for 24 hours. BHA and ATRA were purchased from Sigma and differentiation treatments were maintained for 5 days, and essentially performed as previously indicated (Richter et al., 2009).

Oligofection, transfection and retroviral infection

Oligofection was performed as previously described (Martinez-Romero et al., 2009). To generate retroviral stocks, Phoenix cells were transfected with Fugene (Roche). For stable expression of short hairpin RNA (shRNA) cells were transduced with retroviral supernatants in the presence of Polybrene (4 μg/ml; Sigma) and selected with 2 μg/ml Puromycin (Sigma). Retroviral constructs carrying shRNA sequences with Puromycin resistance were essentially made as previously described (Brummelkamp et al., 2002). Mock, RING1B, BMI1 and EZH2 shRNA sequences are available upon request. Sequences for shRING1B #2 and siFLI1 have been reported before (Wang et al., 2004, and Siligan et al., 2005).

Protein extraction and western blot

These protocols have been performed following standard techniques. RING1B, BMI1, EZH2 antibodies were the same as used for immunohistochemistry. FLI1 antibody was from Santa Cruz (SC356). Anti-$Na_V1.6$ was from Alomone Labs (ASC-009). Cleaved PARP1 and NF-kB proteins were detected using antibodies from Cell Signaling (#5625 and #4766, respectively). Lamin B1 antibody was from Abcam (AB16048) and Tubulin and Actin antibodies form Sigma (T6199 and A2228, respectively). Secondary HRP-conjugated antibodies were obtained from Dako.

Western blot quantification was performed using Image J densitometry software. The intensity of individual bands was normalized to Lamin B or Actin signal, as a measure of protein relative abundance in the different samples and referred to control conditions (arbitrarily set to 100).

RNA isolation and qRT-PCR

RNA was isolated with Genelute Total Mammalian RNA Kit (Sigma) and cDNA was obtained by using Transcriptor First Strand cDNA Synthesis Kit (Roche). qRT-PCR assays were performed using SYBR Green PCR master mix (Applied Biosystems, Life Technologies). For normalization purposes, we ran simultaneously qRT-PCR with primers for GAPDH. The ABI PRISM 7900HT cycler's software calculated a threshold cycle number (Ct) at which each PCR amplification reached a significant threshold level. The figures present the amount of target mRNA/GAPDH mRNA relative copies ratio. Primers used for qRT-PCR were:

	Forward	Reverse
RING1B	CAGACAAACGGAACTCAACCATT	CTGTTATTGCCTCCTGAGGTGTT
EZH2	TTGAACCTCCTGAGAATG	TGTCATAGTAAGTGCCAAT
BMI1	CCAGCGGTAACCACCAATCT	CTTAACAGTCTCAGGTATCAACCAGAA
SCN8A	GGGCGGAAGGACAGAATCAA	CAGGACGATGCAGATGGTGA
FGF14	TGGCCGAAAACAAACAATCACT	GCAGCTTAGACACCCTGAGA
GFAP	TGGGAGCTTGATTCTCAGCA	CCTGGGCTTGACCTCTCTGTA
NF68	GATGTACATGGTAAGTCTCAAACAGAT	TTTTACTATTTTATTATGGCACACAGG
S100B	GAGTTCTTTGAACATGAGTGAGATTAG	AAATCAAGCTTCCTAATTAGCTACAAC
ALCAM	TCAATTGTTTATATGGATAATCTGAGC	ACAGTTTTTAATTAGGGCAATATGAGT
VEGFB	GTACTGTCTCAGTTTCTAACCACTCTG	GGGTCACAGTTCTTGTACCAAAG
GAPDH	AGTCAGCCGCATCTTCTTTTG	AAATCCGTTGACTCCGACCTT

Gene expression analysis

Microarray analysis, amplification, labeling and hybridizations were performed according to protocols from Ambion WT Expression Kit (Ambion), labeled using the WT Terminal Labeling Kit (Affymetrix), and then hybridized to GeneChip Human Gene 2.0 ST Array (Affymetrix) in a GeneChip Hybridization Oven 640. Washing and scanning were performed using the Hybridization Wash and Stain Kit and the GeneChip System of Affymetrix (GeneChip Fluidics Station 450 and GeneChip Scanner 3000 7G). After quality control of raw data, they were background corrected, quantile-normalized and summarized to a gene-level using the robust multi-chip average (RMA) (Irizarry et al., 2003) obtaining a total of 48144 transcript clusters, excluding controls, which roughly correspond to genes and other RNAs, like lincRNAs and miRNAs. NetAffx 33 annotations, human genome 19 built, were used to summarize data into transcript clusters and to annotate analyzed data. Linear Models for Microarray (LIMMA)(Smyth, 2004), a moderated t-statistics model, was used for detecting differentially expressed genes between the conditions. Genes with a p-value less than 0.05 were selected as significant.

All data analysis were performed in R (version 3.1.1) with packages aroma.affymetrix (Bengtsson et al., 2008), Biobase, Affy, limma, genefilter, gplots and Vennerable. Ingenuity Pathway Analysis (Ingenuity® Systems, www.ingenuity.com) was used to perform functional analysis of the results.

ChIP-qPCR analysis

The ChIP-qPCR assays were performed as previously described (Sanchez-Molina et al., 2014). A673 were treated with 1% formaldehyde at room temperature for 10 min and washed once in ice-cold PBS. The pellet was resuspended in lysis buffer (0.1% SDS, 0.15 M NaCl, 1% Triton X-100, 1 mM EDTA, 20 mM Tris pH 8 and 1 mg/ml protease inhibitors) and sonicated with Bioruptor until cross-linked chromatin was sheared to an average DNA fragment length of 0.5 kbp. After centrifugation (30 min at 14,000 rpm), chromatin preparations were precleared by incubation with 40 µl of Protein A agarose/Salmon Sperm DNA 50% gel slurry (Millipore) for 2 h at 4°C under rotation. The protein A-agarose was removed by centrifugation and the pre-cleared chromatin was immunoprecipitated by incubation with 5 µg of the antibody and 50 µl of Protein A agarose O/N at 4 °C. The immunoprecipitates were washed with buffers TSE I (0.1% SDS, 1% Triton X-100, 2Mm EDTA, 20 mM Tris-HCl, pH 8, 150 mM NaCl), TSE II (0.1% SDS, 1% Triton X-100, 2 mM EDTA, 20 mM Tris-HCl, pH 8, 500 mM NaCl) and TSEIII (0.25 M LiCl, 1% Nonidet P-40, 1% deoxycholate, 1 mM EDTA, 10 mM Tris-HCl, pH 8) and three times with Tris-EDTA buffer. Washed pellets were eluted with 120 µl of a solution containing 1% SDS, 0.1 M NaHCO3. Eluted pellets were de-cross-linked with O/N at 65 °C and purified on 50 µl of Tris-EDTA buffer with QIAquick PCR Purification Kit (Qiagen). Differences in the DNA content from every immunoprecipitation assay were determined by real-time PCR using the ABI 7700 sequence detection system and SYBR Green master mix protocol (Applied Biosystems). Every immunoprecipitation was done in duplicate and PCRs were carried out in triplicate using fixed amounts of input and inmunoprecipitated DNA from each cell line at 95 °C for 10 min, followed by 40 cycles of 15 s at 95 °C and 1 min at 60 °C. For every amplicon a standard curves was made. The reported data represent real-time PCR values normalized to input DNAs and expressed as percentage (%) of bound/input signal. The SCN8A primer oligonucleotide sequences are:#1 (forw: CGAGAACACTGAGGTTTGGA; rev: CTCGCGAGGATCTAGGTAATG); #2 (forw: CGCT GATTGACTTCTCGTTT; rev: GCGTCAGTGCCAAG TCTTAC).

Electrophysiology

Series resistance was compensated by 80-90%. Currents were acquired at 33 kHz. The pClamp8 software (Axon Instruments) was used for pulse generation, data acquisition and subsequent analysis. Borosilicate glass patch pipettes had a tip resistance of 2-3 MΩ and were filled with a solution containing (in mM): 140 CsCl, 1 EGTA, 4 Na_2ATP, 0,1 Na_3GTP and 10 Hepes (pH 7.2-7.3 and 295-300 mosmoles/l). The external solution contained the following (in mM): 140 NaCl, 3 CsCl, 1.5 $MgCl_2$ and 10 HEPES, pH 7.3-7.4 (adjusted to 300-305 mosmoles/l). All experiments were performed at room temperature (22-24°C). Data are expressed as mean ± SEM.

For the generation of current-voltage (I-V) curves, current values were divided by the cell capacitance, as read from the amplifier, to obtain the shown current densities at the indicated voltages. In order to obtain the voltage-dependence of channel activation, I-V relationships were fitted with the modified Boltzmann equation:

$$I = \frac{Gmax(V - Vrev)}{1 + e^{\frac{-(V - V1/2\ act)}{kact}}}$$

where I is the peak current, G_{max} is the maximum conductance of the cell, V is the membrane potential, V_{rev} is the extrapolated reversal potential of the current, $V_{1/2act}$ is the voltage for half-maximal current activation, and k_{act} is the slope factor of the Boltzmann term.

Cell migration analysis

Cells were seeded on 24-well plates and grown to confluence. We used a small plastic tip to wound the monolayer at the center of the well prior to imaging using a Zeiss Cell Observer microscope equipped with a 10x objective and controlled temperature (37°C) and atmosphere (5% CO_2) every 30min for 24h. For each well, we tracked a minimum of 6 cells with the MTrackJ plugin of Image J.

SUPPLEMENTAL REFERENCES

1. Richter GH, Plehm S, Fasan A, Rossler S, Unland R, Bennani-Baiti IM, Hotfilder M, Lowel D, von Luettichau I, Mossbrugger I, Quintanilla-Martinez L, Kovar H, Staege MS, et al. EZH2 is a mediator of EWS/FLI1 driven tumor growth and metastasis blocking endothelial and neuro-ectodermal differentiation. Proc Natl Acad Sci U S A. 2009; 106: 5324-9.
2. Wang H, Wang L, Erdjument-Bromage H, Vidal M, Tempst P, Jones RS, and Zhang Y. Role of histone H2A ubiquitination in Polycomb silencing. Nature. 2004; 431: 873-8.
3. Martinez-Romero C, Rooman I, Skoudy A, Guerra C, Molero X, Gonzalez A, Iglesias M, Lobato T, Bosch A, Barbacid M, Real FX, and Hernandez-Munoz I. The epigenetic regulators Bmi1 and Ring1B are differentially regulated in pancreatitis and pancreatic ductal adenocarcinoma. J Pathol. 2009; 219: 205-13.
4. Brummelkamp TR, Bernards R, and Agami R. A system for stable expression of short interfering RNAs in mammalian cells. Science. 2002; 296: 550-3.
5. Siligan C, Ban J, Bachmaier R, Spahn L, Kreppel M, Schaefer KL, Poremba C, Aryee DN, and Kovar H. EWS-FLI1 target genes recovered from Ewing's sarcoma chromatin. Oncogene. 2005; 24: 2512-24.
6. Irizarry RA, Bolstad BM, Collin F, Cope LM, Hobbs B, and Speed TP. Summaries of Affymetrix GeneChip probe level data. Nucleic Acids Res. 2003; 31: e15.
7. Smyth GK. Linear models and empirical bayes methods for assessing differential expression in microarray experiments. Stat Appl Genet Mol Biol. 2004; 3: Article3.
8. Bengtsson H, Simpson K, Bullard J, and Hansen K. Aroma. affymetrix: A generic framework in R for analyzing small to very large Affymetrix data sets in bounded memory. 745. 2008. Department of Statistics, University of California, Berkeley.
9. Sanchez-Molina S, Estaras C, Oliva JL, Akizu N, Asensio-Juan E, Rojas JM, and Martinez-Balbas, MA. Regulation of CBP and Tip60 coordinates histone acetylation at local and global levels during Ras-induced transformation. Carcinogenesis. 2014; 35:2194-202.

A.

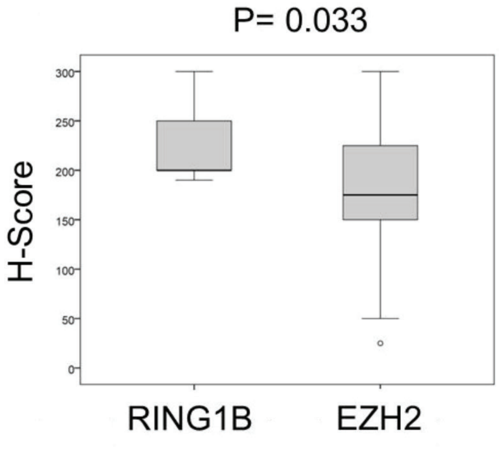

B.

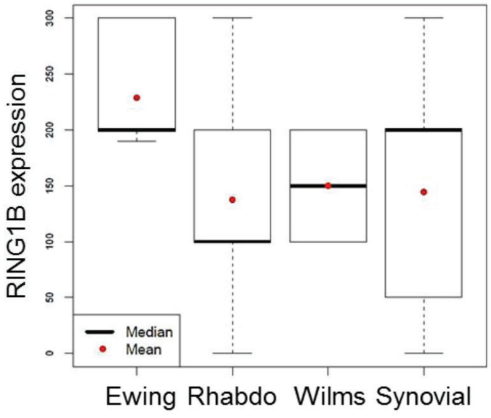

Comparison	Adjusted *p*-value
Ewing sarcoma vs. Rhabdomyosarcoma	0.003
Ewing sarcoma vs. Synovial sarcoma	0.014
Ewing sarcoma vs. Wilms tumor	0.020
Rhabdomyosarcoma vs. Synovial sarcoma	1.000
Rhabdomyosarcoma vs. Wilms tumor	1.000
Synovial sarcoma vs. Wilms tumor	1.000

C.

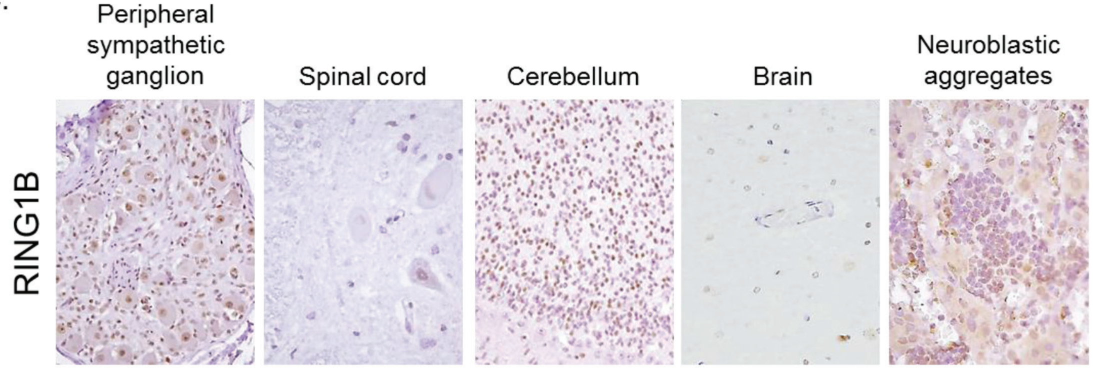

Supplementary Figure S1, related to Figure 1: RING1B expression in Ewing sarcoma and normal tissues. A. Statistical analysis of semiquantitative histoscore evaluation of RING1B and EZH2 signal primary ES tumors. Statistical analysis was performed with the Wilcoxon signed rank test, and figure shows boxplot representation of histoscore values of RING1B and EZH2 expression in ES primary tumors (n=16). **B.** Left, boxplot representation of RING1B expression in Ewing sarcoma (n=17), Rhabdomyosarcoma (n=16), Wilms tumor (n=27) and Synovial sarcoma (n=10). Right, multiple comparison analysis of RING1B expression in these tumors, performed with the Holm-Bonferroni method. **C.** RING1B expression in neural embryonic tissues and neuroblastic aggregates.

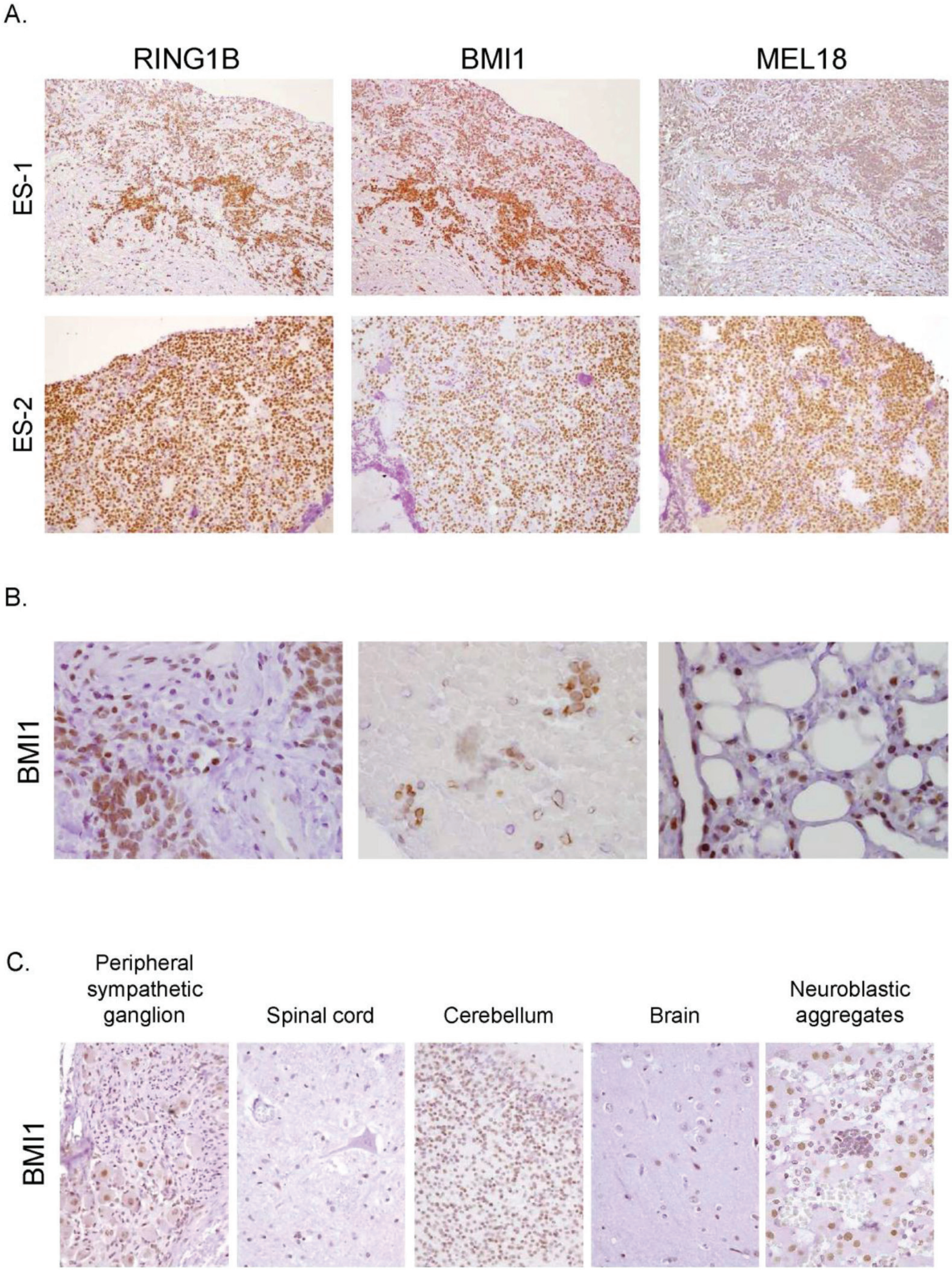

Supplementary Figure S2, related to Figure 1: PRC1 expression in Ewing sarcoma and normal tissues. A. Expression of the PRC1 proteins RING1B, BMI1 and MEL18 in serial sections of two primary ES tumors. **B.** Details of BMI1 expression in a primary ES tumor (left), in a blood clot of an ES tumor (middle) and in adipose tissue of an ES tumor (right). **C.** BMI1 expression in neural tissues and neuroblastic aggregates.

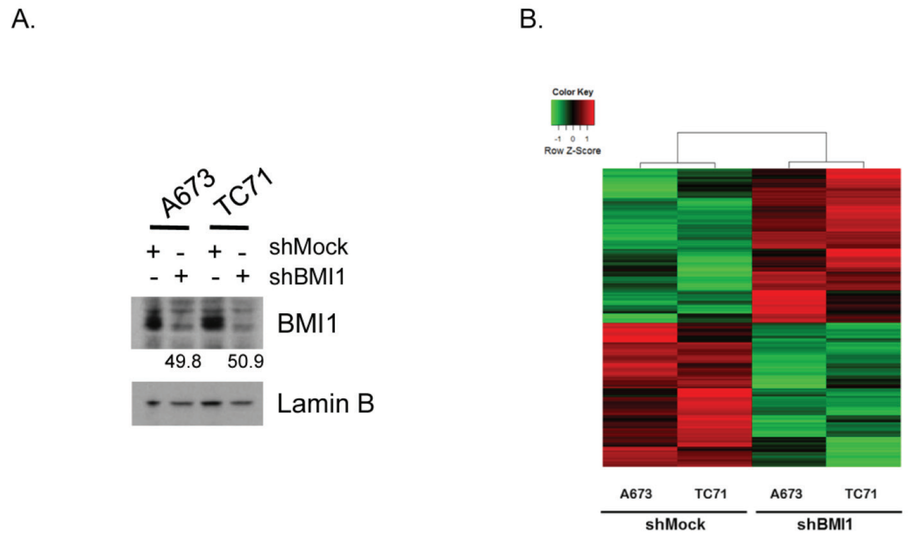

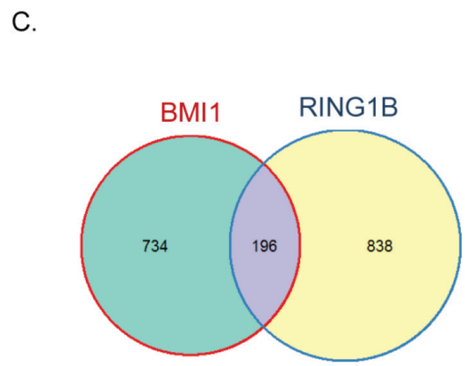

MIR4279	LOC100293977	OR8J1
OR3A2	HLA-C	LOC100506372
GSTA2	CCDC172	NTM-IT1
IGKV6-21	HLA-C	LOC100652750
IGHG1	MIR4642	SERP2
IGKC	IGHV3-35	CABP5
RNU7-19P	CCDC30	USP37
RN5S205	ARHGEF33	CYP2G1P
KRTAP13-1	CDRT1	CYP4F8
MIR320D1	METTL21CP1	DNAJA1P5
FAM194A	MIR561	KLK2
TDRD9	HLA-C	OR2V2
KIAA1755	RNY1P4	EFHA2
PPP1R11	DCDC1	LOC157860
MIR320D2	LOC285593	RNU5A-5P
FCGR1C	TRAJ18	DISP1
TAP2	LOC100287509	MIR3164
RN5S498	RNU6ATAC4P	ARGFXP2
RN5S70	MIR549	GOLGA8DP
C1orf111	LOC283352	MIR1284
MIR573	ACTR3BP2	LOC100506835
PTPRT	OR4A16	ZNF425
LINC00311	DYNLRB2	
IGHA1	KRT23	

Supplementary Figure S3, related to Figure 3: Comparison of BMI1 and RING1B transcriptomes in Ewing sarcoma cells. A. Efficency of stable BMI1 depletion, assessed by immunoblot, in A673 and TC71 cell lines. **B.** Primary heat map of genes differentially expressed between shMock and shBMI1 cells. **C.** Venn diagram of transcripts affected by BMI1 and RING1B depletion in A673 and TC71 cell lines (left) and annotated names of these transcripts (right). Array data was deposited at the Gene Expression Omnibus (GEO, National Center for Biotechnology Information) with accession number GSE71007.

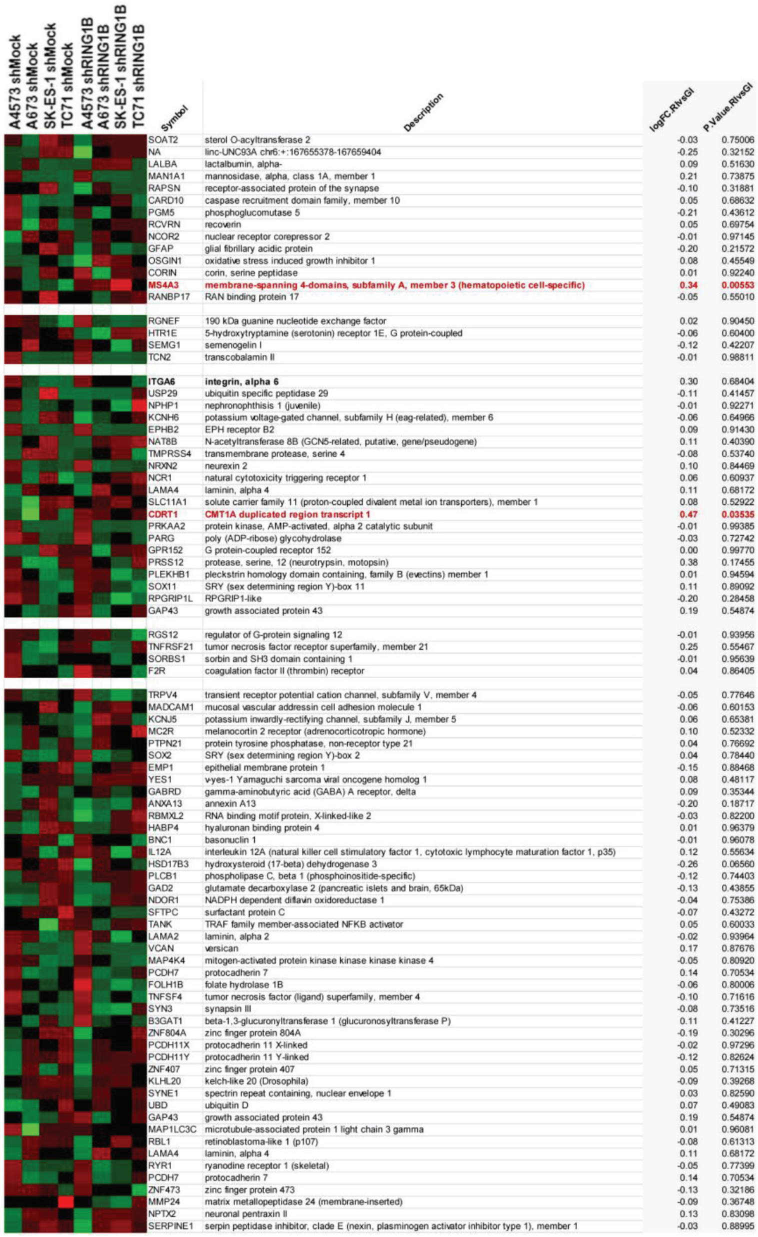

Symbol	Description	logFC.RIvsGI	P.Value.RIvsGI
SOAT2	sterol O-acyltransferase 2	-0.03	0.75006
NA	linc-UNC93A chr6:+:167655378-167659404	-0.25	0.32152
LALBA	lactalbumin, alpha-	0.09	0.51630
MAN1A1	mannosidase, alpha, class 1A, member 1	0.21	0.73875
RAPSN	receptor-associated protein of the synapse	-0.10	0.31881
CARD10	caspase recruitment domain family, member 10	0.05	0.68632
PGM5	phosphoglucomutase 5	-0.21	0.43612
RCVRN	recoverin	0.05	0.69754
NCOR2	nuclear receptor corepressor 2	-0.01	0.97145
GFAP	glial fibrillary acidic protein	-0.20	0.21572
OSGIN1	oxidative stress induced growth inhibitor 1	0.08	0.45549
CORIN	corin, serine peptidase	0.01	0.92240
**MS4A3**	**membrane-spanning 4-domains, subfamily A, member 3 (hematopoietic cell-specific)**	**0.34**	**0.00553**
RANBP17	RAN binding protein 17	-0.05	0.55010
RGNEF	190 kDa guanine nucleotide exchange factor	0.02	0.90450
HTR1E	5-hydroxytryptamine (serotonin) receptor 1E, G protein-coupled	-0.06	0.60400
SEMG1	semenogelin I	-0.12	0.42207
TCN2	transcobalamin II	-0.01	0.98811
**ITGA6**	**integrin, alpha 6**	0.30	0.68404
USP29	ubiquitin specific peptidase 29	-0.11	0.41457
NPHP1	nephronophthisis 1 (juvenile)	-0.01	0.92271
KCNH6	potassium voltage-gated channel, subfamily H (eag-related), member 6	-0.06	0.64966
EPHB2	EPH receptor B2	0.09	0.91430
NAT8B	N-acetyltransferase 8B (GCN5-related, putative, gene/pseudogene)	0.11	0.40390
TMPRSS4	transmembrane protease, serine 4	-0.08	0.53740
NRXN2	neurexin 2	0.10	0.84469
NCR1	natural cytotoxicity triggering receptor 1	0.06	0.60937
LAMA4	laminin, alpha 4	0.11	0.68172
SLC11A1	solute carrier family 11 (proton-coupled divalent metal ion transporters), member 1	0.08	0.52922
**CDRT1**	**CMT1A duplicated region transcript 1**	**0.47**	**0.03535**
PRKAA2	protein kinase, AMP-activated, alpha 2 catalytic subunit	-0.01	0.99385
PARG	poly (ADP-ribose) glycohydrolase	-0.03	0.72742
GPR152	G protein-coupled receptor 152	0.00	0.99770
PRSS12	protease, serine, 12 (neurotrypsin, motopsin)	0.38	0.17455
PLEKHB1	pleckstrin homology domain containing, family B (evectins) member 1	0.01	0.94594
SOX11	SRY (sex determining region Y)-box 11	0.11	0.89092
RPGRIP1L	RPGRIP1-like	-0.20	0.28458
GAP43	growth associated protein 43	0.19	0.54874
RGS12	regulator of G-protein signaling 12	-0.01	0.93956
TNFRSF21	tumor necrosis factor receptor superfamily, member 21	0.25	0.55467
SORBS1	sorbin and SH3 domain containing 1	-0.01	0.95639
F2R	coagulation factor II (thrombin) receptor	0.04	0.86405
TRPV4	transient receptor potential cation channel, subfamily V, member 4	-0.05	0.77646
MADCAM1	mucosal vascular addressin cell adhesion molecule 1	-0.06	0.60153
KCNJ5	potassium inwardly-rectifying channel, subfamily J, member 5	0.06	0.65381
MC2R	melanocortin 2 receptor (adrenocorticotropic hormone)	0.10	0.52332
PTPN21	protein tyrosine phosphatase, non-receptor type 21	0.04	0.76692
SOX2	SRY (sex determining region Y)-box 2	0.04	0.78440
EMP1	epithelial membrane protein 1	-0.15	0.88468
YES1	v-yes-1 Yamaguchi sarcoma viral oncogene homolog 1	0.08	0.48117
GABRD	gamma-aminobutyric acid (GABA) A receptor, delta	0.09	0.35344
ANXA13	annexin A13	-0.20	0.18717
RBMXL2	RNA binding motif protein, X-linked-like 2	-0.03	0.82200
HABP4	hyaluronan binding protein 4	0.01	0.96379
BNC1	basonuclin 1	-0.01	0.96078
IL12A	interleukin 12A (natural killer cell stimulatory factor 1, cytotoxic lymphocyte maturation factor 1, p35)	0.12	0.55634
HSD17B3	hydroxysteroid (17-beta) dehydrogenase 3	-0.26	0.06560
PLCB1	phospholipase C, beta 1 (phosphoinositide-specific)	-0.12	0.74403
GAD2	glutamate decarboxylase 2 (pancreatic islets and brain, 65kDa)	-0.13	0.43855
NDOR1	NADPH dependent diflavin oxidoreductase 1	-0.04	0.75386
SFTPC	surfactant protein C	-0.07	0.43272
TANK	TRAF family member-associated NFKB activator	0.05	0.60033
LAMA2	laminin, alpha 2	-0.02	0.93964
VCAN	versican	0.17	0.87676
MAP4K4	mitogen-activated protein kinase kinase kinase kinase 4	-0.05	0.80920
PCDH7	protocadherin 7	0.14	0.70534
FOLH1B	folate hydrolase 1B	-0.06	0.80006
TNFSF4	tumor necrosis factor (ligand) superfamily, member 4	-0.10	0.71616
SYN3	synapsin III	-0.08	0.73516
B3GAT1	beta-1,3-glucuronyltransferase 1 (glucuronosyltransferase P)	0.11	0.41227
ZNF804A	zinc finger protein 804A	-0.19	0.30296
PCDH11X	protocadherin 11 X-linked	-0.02	0.97296
PCDH11Y	protocadherin 11 Y-linked	-0.12	0.82624
ZNF407	zinc finger protein 407	0.05	0.71315
KLHL20	kelch-like 20 (Drosophila)	-0.09	0.39268
SYNE1	spectrin repeat containing, nuclear envelope 1	0.03	0.82590
UBD	ubiquitin D	0.07	0.49083
GAP43	growth associated protein 43	0.19	0.54874
MAP1LC3C	microtubule-associated protein 1 light chain 3 gamma	0.01	0.96081
RBL1	retinoblastoma-like 1 (p107)	-0.08	0.61313
LAMA4	laminin, alpha 4	0.11	0.68172
RYR1	ryanodine receptor 1 (skeletal)	-0.05	0.77399
PCDH7	protocadherin 7	0.14	0.70534
ZNF473	zinc finger protein 473	-0.13	0.32186
MMP24	matrix metallopeptidase 24 (membrane-inserted)	-0.09	0.36748
NPTX2	neuronal pentraxin II	0.13	0.83098
SERPINE1	serpin peptidase inhibitor, clade E (nexin, plasminogen activator inhibitor type 1), member 1	-0.03	0.88995

Supplementary Figure S4, related to Figure 4: Transcriptional status of the EZH2-targets identified by Richter *et al.* (2009, Table 1) in Ewing sarcoma cell lines. Heat map representation of microarray expression data from RING1B depleted cells of genes identified as altered by EZH2 knockdown.

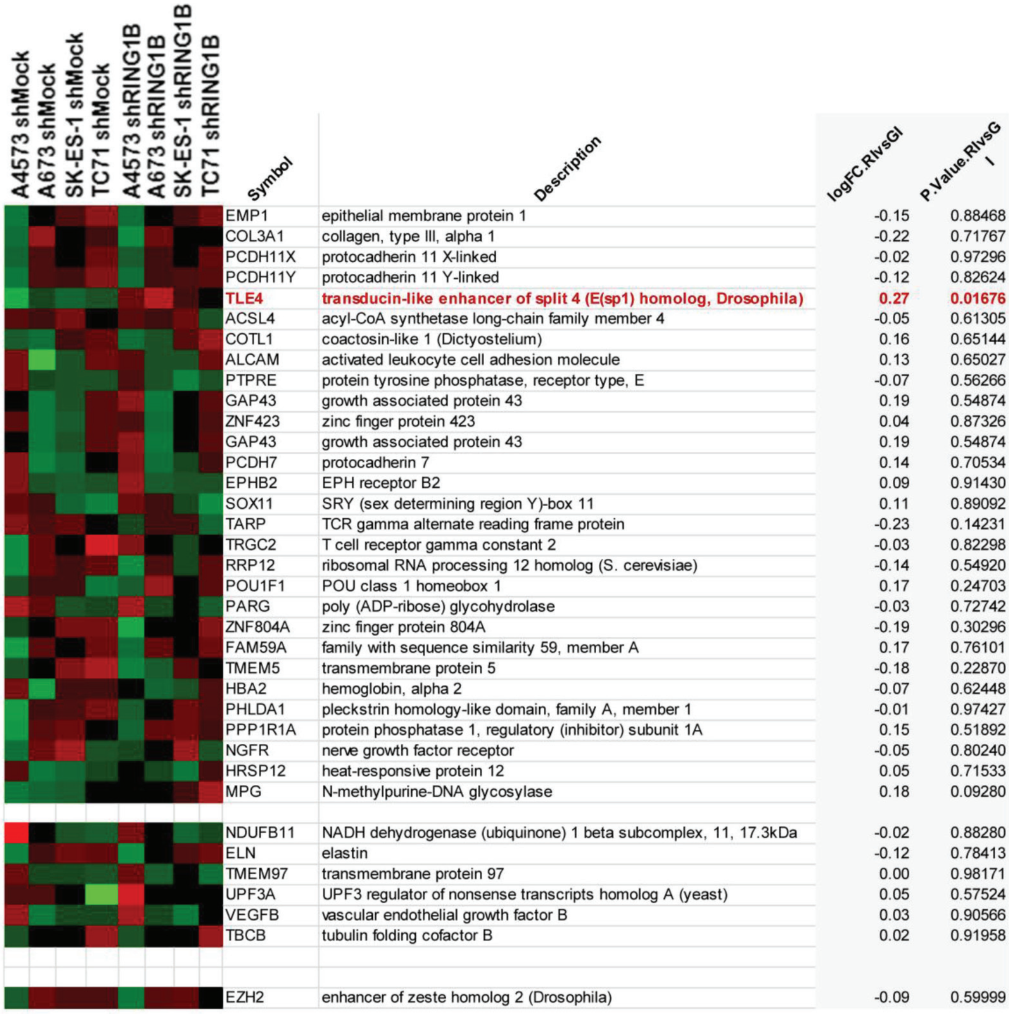

Symbol	Description	logFC.RIvsGI	P.Value.RIvsGI
EMP1	epithelial membrane protein 1	-0.15	0.88468
COL3A1	collagen, type III, alpha 1	-0.22	0.71767
PCDH11X	protocadherin 11 X-linked	-0.02	0.97296
PCDH11Y	protocadherin 11 Y-linked	-0.12	0.82624
**TLE4**	**transducin-like enhancer of split 4 (E(sp1) homolog, Drosophila)**	**0.27**	**0.01676**
ACSL4	acyl-CoA synthetase long-chain family member 4	-0.05	0.61305
COTL1	coactosin-like 1 (Dictyostelium)	0.16	0.65144
ALCAM	activated leukocyte cell adhesion molecule	0.13	0.65027
PTPRE	protein tyrosine phosphatase, receptor type, E	-0.07	0.56266
GAP43	growth associated protein 43	0.19	0.54874
ZNF423	zinc finger protein 423	0.04	0.87326
GAP43	growth associated protein 43	0.19	0.54874
PCDH7	protocadherin 7	0.14	0.70534
EPHB2	EPH receptor B2	0.09	0.91430
SOX11	SRY (sex determining region Y)-box 11	0.11	0.89092
TARP	TCR gamma alternate reading frame protein	-0.23	0.14231
TRGC2	T cell receptor gamma constant 2	-0.03	0.82298
RRP12	ribosomal RNA processing 12 homolog (S. cerevisiae)	-0.14	0.54920
POU1F1	POU class 1 homeobox 1	0.17	0.24703
PARG	poly (ADP-ribose) glycohydrolase	-0.03	0.72742
ZNF804A	zinc finger protein 804A	-0.19	0.30296
FAM59A	family with sequence similarity 59, member A	0.17	0.76101
TMEM5	transmembrane protein 5	-0.18	0.22870
HBA2	hemoglobin, alpha 2	-0.07	0.62448
PHLDA1	pleckstrin homology-like domain, family A, member 1	-0.01	0.97427
PPP1R1A	protein phosphatase 1, regulatory (inhibitor) subunit 1A	0.15	0.51892
NGFR	nerve growth factor receptor	-0.05	0.80240
HRSP12	heat-responsive protein 12	0.05	0.71533
MPG	N-methylpurine-DNA glycosylase	0.18	0.09280
NDUFB11	NADH dehydrogenase (ubiquinone) 1 beta subcomplex, 11, 17.3kDa	-0.02	0.88280
ELN	elastin	-0.12	0.78413
TMEM97	transmembrane protein 97	0.00	0.98171
UPF3A	UPF3 regulator of nonsense transcripts homolog A (yeast)	0.05	0.57524
VEGFB	vascular endothelial growth factor B	0.03	0.90566
TBCB	tubulin folding cofactor B	0.02	0.91958
EZH2	enhancer of zeste homolog 2 (Drosophila)	-0.09	0.59999

Supplementary Figure S5, related to Figure 4: Transcriptional status of the EZH2-targets identified by Richter et al. (2009, Table 2) in Ewing sarcoma cell lines. Heat map representation of microarray expression data from RING1B depleted cells of genes identified as altered by EZH2 knockdown.

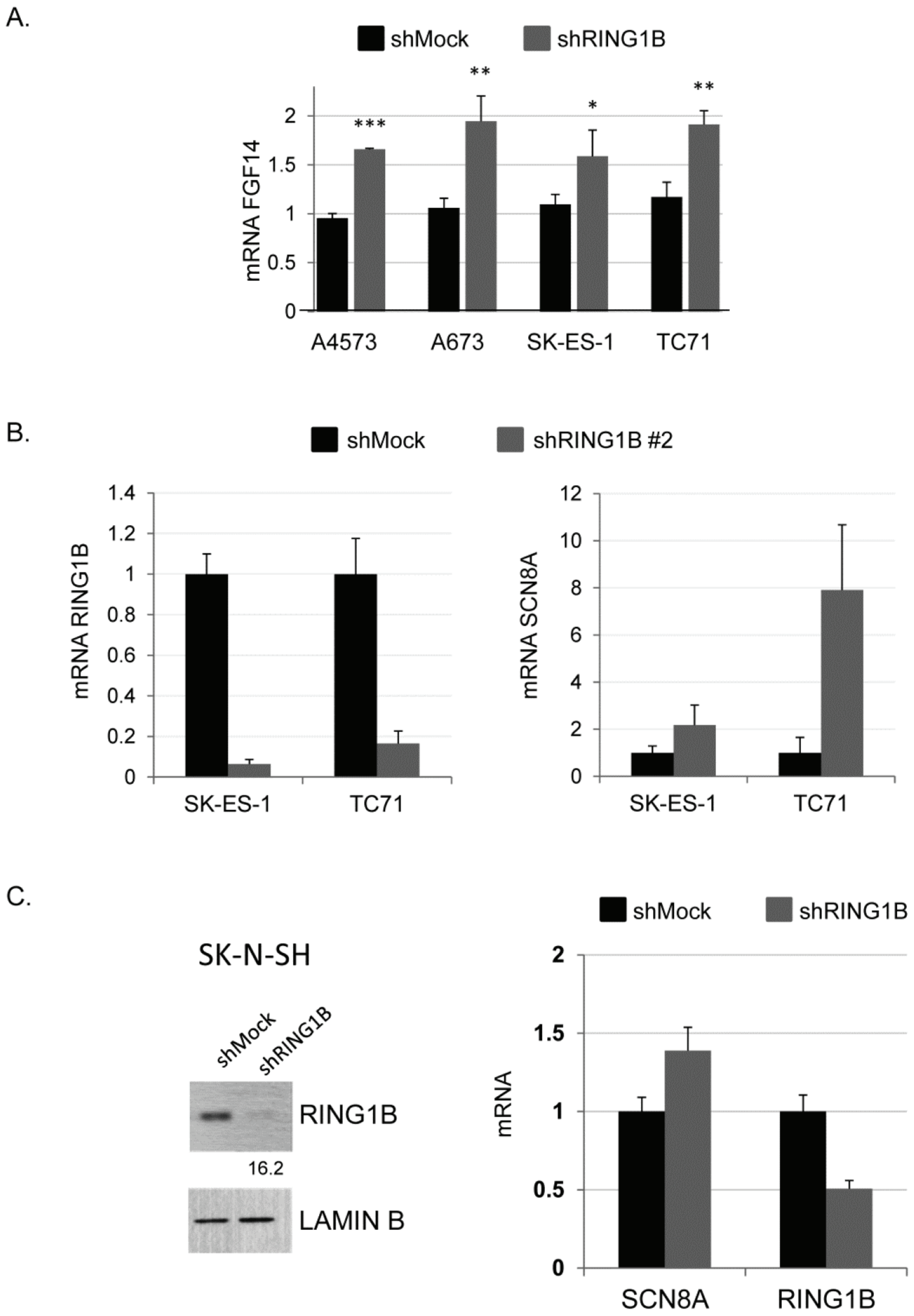

Supplementary Figure S6, related to Figure 5: Effects of RING1B stable depletion in ES cell lines and SK-N-SH (neuroblastoma) cell lines. A. FGF14 mRNA levels in RING1B depleted cells determined by qRT-PCR and referred to their own shMock control mRNA levels. *, $P < 0.05$; **, $P < 0.01$; ***, $P < 0.005$. **B.** RING1B and SCN8A mRNA levels in stably RING1B-depleted cells with an independent shRNA sequence (Wang et al., 2004), determined by qRT-PCR and referred to their shMock control mRNA levels. **C.** Efficiency of stable RING1B depletion in SK-N-SH (neuroblastoma) cells, analyzed by immunoblot (left panel) and SCN8A and RING1B mRNA levels, determined by qRT-PCR. Graph shows mean ± SD of one representative experiment performed in triplicate.

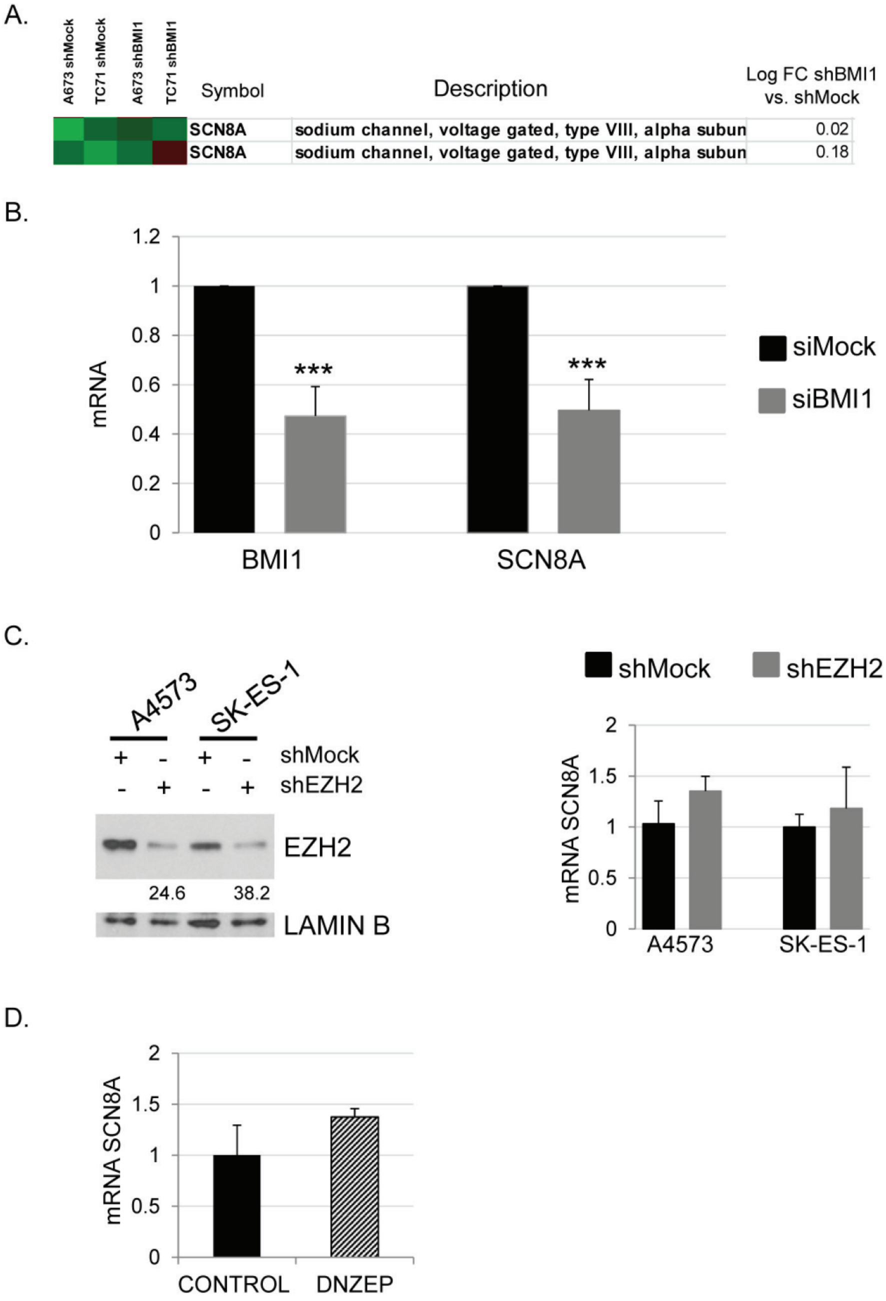

Supplementary Figure S7, related to Figure 5: Effect of BMI1 and EZH2 depletion on SCN8A expression in Ewing sarcoma cell lines. A. Heat map representation of SCN8A expression in A673 and TC-71 BMI1-depleted cells. **B.** Analysis of BMI1 and SCN8A mRNA levels in A673 transiently transfected with Mock or BMI1 siRNAs, determined by qRT-PCR. Mean ± SD of three independent experiments performed in triplicate. **C.** Efficiency of stable EZH2 depletion in A4573 and SK-ES-1 Ewing sarcoma cell lines, determined by immunoblot (left panel) and SCN8A mRNA levels in EZH2 depleted cells, analyzed by qRT-PCR (right panel). **D.** SCN8A mRNA levels detected by qRT-PCR in A673 cells upon 24h treatment with DNZep (5μM). Mean ± SD of one representative experiment performed in triplicate.

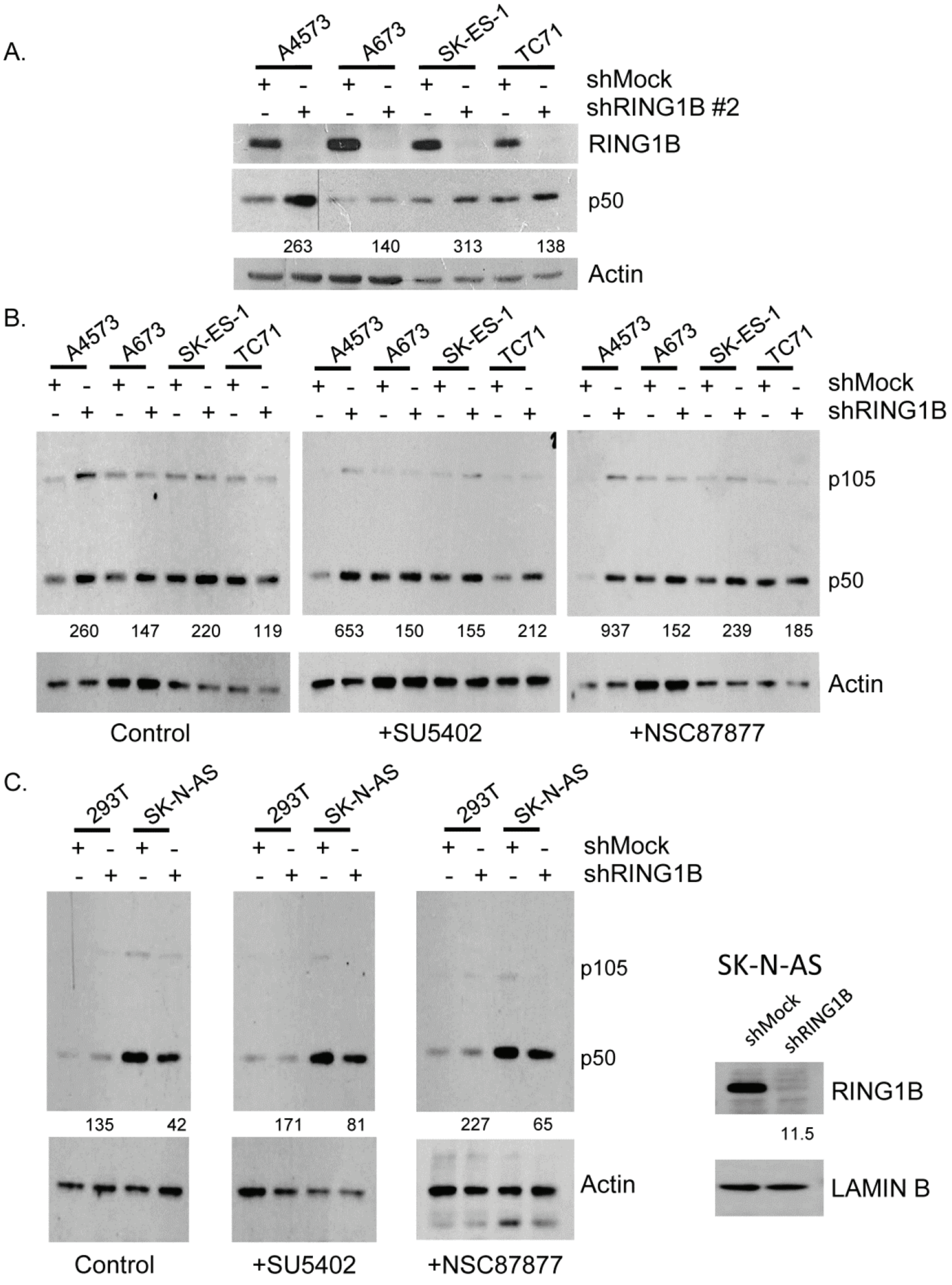

Supplementary Figure S8, related to Figures 6 and 7: NF-kB1 levels in RING1B depleted cells. A. Levels of the NF-κB p50 subunit, detected by immunoblot in shRING1B cells stablished with an independent shRNA sequence (Wang et al., 2004). Numbers at the bottom, band intensities normalized to Actin and relative to their own control cells. **B.** NF-kB1 levels detected by immunoblot in shMock and shRING1B cells untreated (left), or treated with the FGFR inhibitor SU5402 (25µM, middle panel) or the SHP2 phosphatase inhibitor NSC87877 (50µM, right panel). **C.** NF-kB1 levels detected by immunoblot in shMock and shRING1B 293T and SK-N-AS cells, treated as in A. Efficiency of RING1B depletion in SK-N-AS, right panel.
